# Wnt inhibition alleviates resistance to anti-PD1 therapy and improves antitumor immunity in glioblastoma

**DOI:** 10.1073/pnas.2414941122

**Published:** 2025-09-15

**Authors:** Shanmugarajan Krishnan, Somin Lee, Zohreh Amoozgar, Sonu Subudhi, Ashwin Srinivasan Kumar, Jessica M. Posada, Neal Lindeman, Pinji Lei, Mark Duquette, Sophie Steinbuch, Marc Charabati, Peigen Huang, Patrik Andersson, Meenal Datta, Lance L. Munn, Dai Fukumura, Rakesh K. Jain

**Affiliations:** ^a^Department of Radiation Oncology, Edwin L. Steele Laboratories, Massachusetts General Hospital and Harvard Medical School, Boston, MA 02114; ^b^Harvard–Massachusetts Institute of Technology, Division of Health Sciences and Technology, Massachusetts Institute of Technology, Cambridge, MA 02139; ^c^Department of Pathology, Brigham and Women’s Hospital, Boston, MA 02115; ^d^Department of Dermatology, University Medical Center Bonn, Bonn 53127, Germany

**Keywords:** glioblastoma, Wnt7b/β-catenin, immunotherapy, dendritic cells, stem cells

## Abstract

Wnt signaling plays a crucial role for many developmental processes. It is also pivotal in the generation and limited treatment outcomes of glioblastoma (GBM). Here, we identified Wnt7b, which is markedly upregulated in GBM patients, as a determinant of resistance to immune checkpoint blockers (αPD1; anti-Programmed Cell Death Protein 1) in a clinically relevant, αPD1-resistant GBM murine model with abundant stem cells. We observed that increased levels of Wnt7b and β-catenin correlated with the resistance to αPD1. Treatment combining a porcupine inhibitor WNT974 with αPD1 reprogrammed the immune suppressive tumor microenvironment (TME) to bolster antitumor immune responses and extended the survival of mice bearing orthotopic GBM, with 25% long-term survivors. Our causal studies revealed that WNT974 potentiated αPD1 therapy by the expansion of antigen presenting DC3-like dendritic cells (DCs). Additionally, WNT974 combination with αPD1 was associated with a reduction in immune suppressive granulocytic myeloid-derived suppressor cells (MDSCs), an increase in the Ki67+CD8/Ki67+regulatory T cells (Treg) ratio, tilting the CD8:Treg balance in the TME toward antitumor immune response, and more pronounced GrzB+CD8+ effector T cells. Conversely, an increase in monocytic MDSCs and phosphorylation of pro-oncogenic proteins was associated with resistance to the combination therapy. Collectively, our preclinical findings provide a strong rationale to test Wnt7b/β-catenin inhibition with αPD1 therapy in GBM patients with elevated Wnt7b/β-catenin signaling.

## Significance

GBM patients face extremely limited treatment options and poor responses to current immunotherapies. Our findings reveal that targeting the Wnt7b/β-catenin pathway can sensitize stem-cell rich GBM to immune checkpoint blockade, offering a promising therapeutic avenue. We demonstrate the potential of a porcupine inhibitor, WNT974 shown to be safe in a phase I trial in patients with extracranial tumors, to synergize with αPD1 therapy by alleviating immunosuppressive mechanisms and enhancing antitumor immunity. These results support the rationale for personalized clinical trials in glioblastoma patients with elevated Wnt7b/β-catenin signaling, aiming to overcome resistance to immunotherapy. This approach may help improve outcomes for patients with GBM, a disease for which new strategies are urgently needed.

Glioblastoma (GBM) is a fatal malignancy, with a median survival of less than 2 y with current treatments. Immune checkpoint blockers (ICBs) have provided durable responses in several malignancies but have failed in *all* Phase III trials in recurrent and newly diagnosed GBM [(1) NCT02617589] (2, 3). This limited efficacy of ICBs is attributed to i) ICB-induced increase in brain edema that requires the use of immunosuppressive steroids (4), ii) poor infiltration of T cells due to dysfunctional GBM vasculature (5), iii) widespread immunosuppression in the GBM TME caused by infiltration of myeloid-derived cells and regulatory T cells (Tregs) (6–8), iv) deficiency in antigen presentation (9) and v) prevalence of dysfunctional T cells (10). Therefore, new therapeutic strategies are urgently needed to improve immunotherapy outcomes for GBM patients. The Wnt pathway is a key regulator of neural stem cells in embryonic development and adult neurogenesis (11, 12). Wnt signaling is dysregulated in GBM and fuels GBM progression due to its role in proliferation, stemness, and epithelial–mesenchymal transition (EMT) (13–18). Hypermethylation of Wnt signaling repressors is observed in about 40 to 50% of GBM patients (19). Downregulation of Wnt inhibitory factor-1 in 75% of GBM indicates frequent involvement of aberrant Wnt signaling and may render GBM sensitive to inhibitors of Wnt signaling (20, 21). Multiomics analyses in 123 longitudinal GBM pairs identified activation of the Wnt/planar cell polarity pathway and B-Raf proto-oncogene kinase as key molecular drivers of neuronal transition in recurrent GBM (22).

Here, we demonstrate that Wnt signaling is necessary for GBM maintenance and is associated with resistance to αPD1 therapy. Targeting Wnt signaling using a porcupine inhibitor WNT974 alleviates immune suppression in a stem cell–rich, αPD1-resistant murine GBM model in the orthotopic setting and improved survival in combination with αPD1 treatment by remodeling the tumor microenvironment from protumorigenic to antitumorigenic. WNT974 was shown to be safe in a Phase I clinical trial in patients with advanced non-central nervous system (CNS) tumors (23). Moreover, biomarker analyses in this trial suggested that WNT974 could influence immune cell recruitment to these tumors, and may enhance checkpoint inhibitor activity (24). Hence, our preclinical findings provide a powerful rationale for testing Wnt inhibition with αPD1 in GBM patients.

## Results

### Wnt7b Is Upregulated in Human GBMs and Is Essential for the Maintenance of 005GSC—A Stem Cell—Rich Murine GBM Model.

**Fig. 1.**
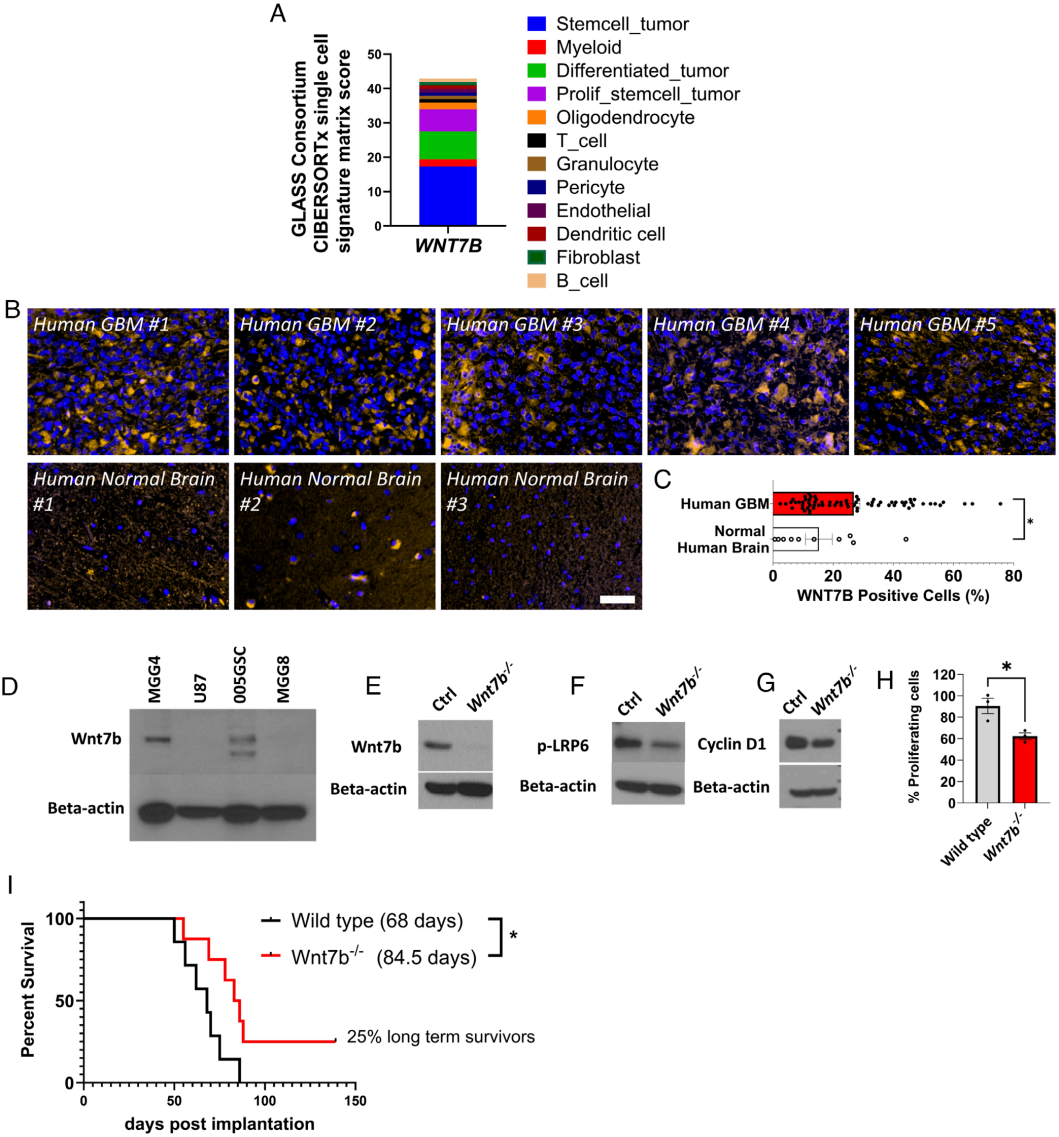
Wnt7b is highly expressed in human GBMs and is essential for the maintenance of 005GSC, a murine model recapitulating stemness in GBM. (*A*) Expression of *WNT7B* obtained by deconvolution of the GLASS gene expression dataset (25) by applying CIBERSORTx (26) using reference cell-state signatures derived from 55, 284 single-transcriptomes from 11 adult patients spanning glioma subtypes and time points (27). (*B*) GBM patient tumor tissues were stained for WNT7B protein using IF. Images of five representative patients from a total of 70 patients and three representative normal cerebrum tissues from a total of 10 normal tissues are shown. DAPI stain is shown in blue and anti-WNT7B positive signaling is shown in orange color. (Scale bar, 50 µM.) (*C*) Quantification of WNT7B positive cells among total cells presented in each patient tissue. *P < 0.05, Two-tailed, Unpaired Student’s *t* test. (n = 70 for GBM patient sample and n = 10 for normal brain tissue). (*D*) WNT7B protein levels were measured in lysates of patient derived GBM cell lines MGG4, U87, and MGG8. Murine 005GSC was used as a positive control for Wnt7b. Western blot analysis of (*E*) Wnt7b and (*F*) pLRP6 and (*G*) Cyclin D1 protein levels in wild type and *Wnt7b^−/−^* cells. Beta-actin was used as a loading control. (*H*) MTT assay performed at 120 h post seeding shows % viable cells. (*I*) 5,000 wild type or *Wnt7b*^−/−^ cells were implanted in immune competent C57BL/6 mice (n = 7 and 8, respectively) orthotopically, and survival was monitored. *P < 0.05, Log-rank (Mantel-Cox) test.

We identified the major Wnt ligands expressed in GBMs in the Glioma Longitudinal Analysis (GLASS) Consortium gene expression dataset composed of 168 GBM patients [121 isocitrate dehydrogenase (IDH) wildtype and 40 IDH mutant] obtained from 37 hospitals worldwide (25). We used CIBERSORTx (26) to deconvolute the GLASS dataset and reference cell-state signatures derived from 55,284 single-transcriptomes from 11 adult patients spanning glioma subtypes and time points (27). This deconvolution revealed *WNT7B, WNT4,* and *WNT6* as the Wnt ligands expressed in 168 patients. *WNT7B* was mostly expressed in stem-cell-rich tumors followed by differentiated tumors and proliferative stem cell tumors (Fig. 1*A*). Based on our TCGA analysis of 223 patients, we stratified GBM tumors into three molecular subtypes—proneural, classical, and mesenchymal—using the Wang et al. classification (28). *WNT7B* expression was lowest in the classical subtype and elevated in both mesenchymal and proneural tumors (*SI Appendix*, Fig. S1*A*). Survival analysis revealed that, among mesenchymal GBMs, patients with higher *WNT7B* expression experienced significantly poorer outcomes than those with lower expression (*SI Appendix*, Fig. S1*B*). Single cell RNA-seq data from 28 IDH-wildtype GBMs showed that *WNT7B* is expressed in malignant cells, macrophages, and CD8 T cells (*SI Appendix*, Fig. S1*C*). Other Wnt genes, including *WNT4* and *WNT6* were mainly expressed in fibroblasts and pericytes (*SI Appendix*, Fig. S1*D*). Furthermore, our integrated analysis of multiple GBM scRNA-seq datasets (29–34) (*SI Appendix*, Fig. S1 *E*–*J*), revealed that *WNT7B* is predominantly expressed in the malignant tumor cell population (*SI Appendix*, Fig. S1 *F* and *G*). Stratification based on M1-like and M2-like gene module scores (*SI Appendix*, Fig. S1 *H* and *I*) shows that patients with higher Wnt7b expression in tumor cells tend to have higher M2-like module scores (*SI Appendix*, Fig. S1*J*). Importantly, *SI Appendix*, Fig. S1*J* reflects patient-level analysis, where both Wnt7b expression and the M1-like/M2-like module ratio were averaged across malignant cells per patient, allowing us to correlate tumor-intrinsic *WNT7B* expression with the macrophage polarization state at the patient level.

WNT7B staining in a tissue array comprising 70 GBM patient tumor tissues and 10 normal cerebrum tissues revealed that GBM tissues had elevated Wnt7b (Fig. 1 *B* and *C*). In addition to the array, we stained for WNT7B protein in 15 GBM biopsies from the Brigham and Women’s Hospital, Boston. All 15 patients showed positive signal from WNT7B immunostaining. Representative images of three patients and the % WNT7B+ cells in whole tumors are shown in *SI Appendix*, Fig. S2 *A* and *B*. Collectively, these results confirm that WNT7B is elevated in certain cohorts of GBM patients. Importantly, a fraction of patients had no or low expression.

Next, we profiled the 19 *Wnt* ligands in murine GBM models that are resistant to αPD1: 005GSC—a stem-cell rich (35, 36), CT-2A—a cell line with antigen presentation deficiency (9, 37), and GL-261-MGH (8) in vitro and found that 005GSC has a higher expression of *Wnt* ligands than the other two lines (*SI Appendix*, Fig. S3*A*). Among the *Wnt* ligands in 005GSC, *Wnt7b*, *Wnt7a,* and *Wnt5a* have the highest expression. We then implanted GBM models (n = 3 per group) in immune competent mice. The messenger RNA (mRNA) expression in vivo was also elevated similar to in vitro levels. Specifically, 005GSC had higher *Wnt5a, Wnt7a, and Wnt7b* expression compared to CT-2A. Note that *Wnt7b* expression is fourfold higher than *Wnt7a* and *Wnt5a* in 005GSC (*SI Appendix*, Fig. S3*B*: y-axis in log scale). In concert with the mRNA data, 005GSC exhibited a high protein level of Wnt7b as compared to CT-2A and GL-261-MGH in vivo (*SI Appendix*, Fig. S3*C*) (n = 3). Wnt5a was not detectable at the protein level.

We next checked Wnt7b levels in human and murine GBM cell lines: U87 (a widely used GBM cell line), two cell lines generated from patients at MGH, MGG4 (stem-like) (38) and MGG8 (diffusely invasive) (38), 005GSC and CT-2A. WNT7B was higher in MGG4 than MGG8 or U87. 005GSC was used as a positive control for Wnt7b (Fig. 1*D*). Collectively, these data demonstrate that Wnt7b expression is elevated in human GBM patients, human GBM cell lines and the murine 005GSC stem-cell rich cell line.

To investigate the causal role of Wnt7b in tumor cell survival in vitro and in vivo in a syngeneic model, we performed CRISPR/Cas9-mediated deletion of *Wnt7b* and clonal selection in 005GSC. Western blot analysis revealed that as compared to the wild type 005GSC, Wnt7b protein was almost absent in *Wnt7b*^−/−^ 005GSC cells, and pLRP6-Ser1490 and Cyclin D1 (a known canonical Wnt target) were lower in the knockout clone (Fig. 1 *E–G*). MTT assay performed at 120 h showed a 30% reduction in proliferation of the *Wnt7b*^−/−^ clone (Fig. 1*H*). We then implanted 5,000 wild type (n = 8) and 5,000 knockout cells (n = 7) orthotopically in mice. The median survival of the 005GSC^*Wnt7bWt*^ –bearing mice was 68 d postimplantation, whereas that of the 005GSC^*Wnt7b−/−*^ –bearing mice was significantly higher at 84.5 d (*P* < 0.05; Fig. 1*I*). Moreover, 25% of the mice from the 005GSC^*Wnt7b−/−*^ group survived more than 135 d (Fig. 1*I*). Cancer stemness is strongly associated with immune suppressive pathway signatures across 21 cancer types, including GBM (39). Intrinsic Wnt pathway elevation is linked to suppression of antitumor immunity in extracranial tumors (40). Therefore, it is plausible that the intrinsic elevation of the Wnt pathway that plays an important role in the maintenance of the stemness in 005GSC is associated with intrinsic resistance to immunotherapies.

### Wnt7b/β-Catenin Elevation Correlates with Adaptive Resistance to αPD1 Therapy.

**Fig. 2.**
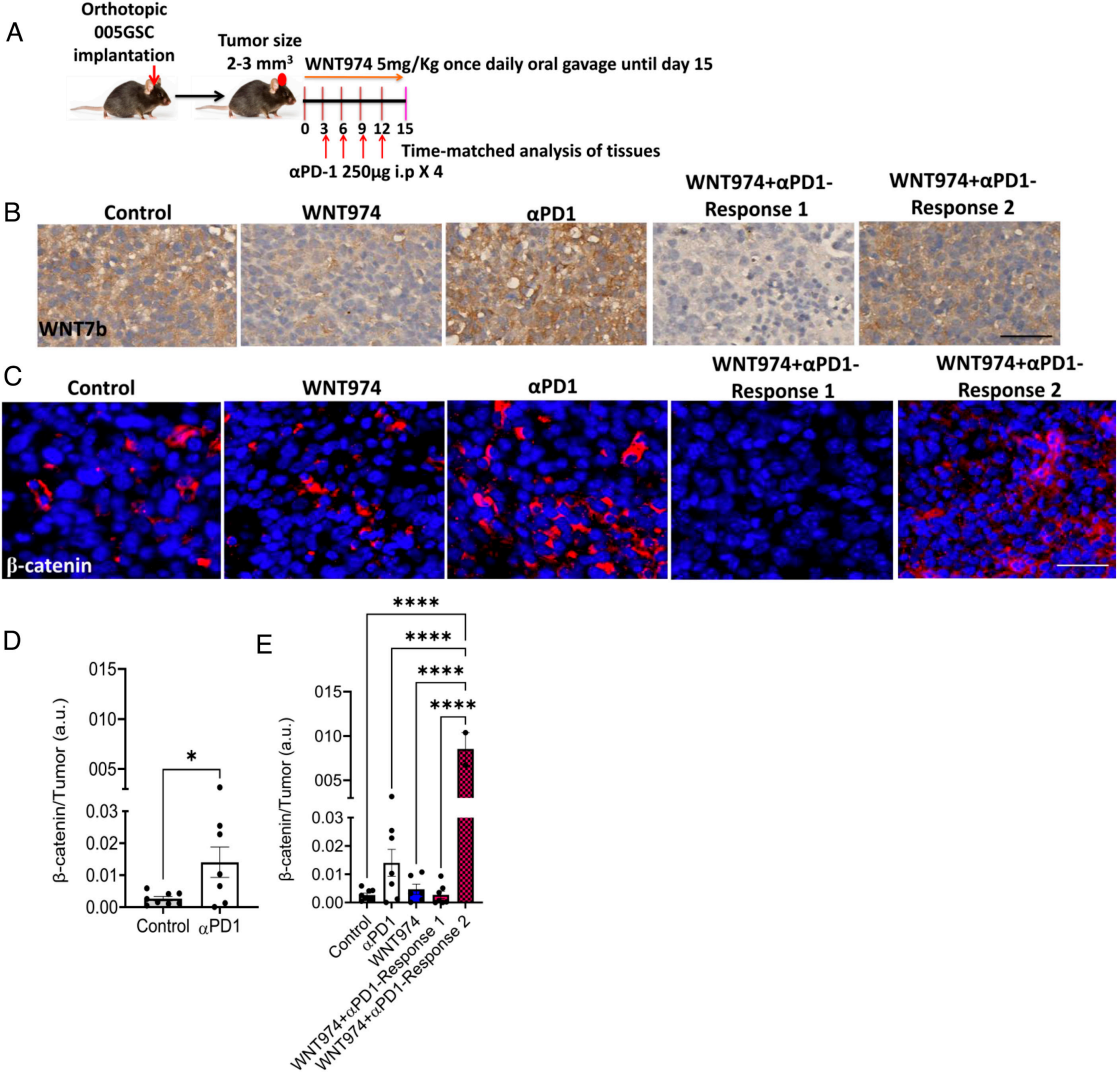
Wnt7b/β-catenin elevation correlates with adaptive resistance to αPD1 therapy. (*A*) C57BL/6 mice were orthotopically implanted with 30,000 005-GSC-GFP. Mice were randomized at 2 to 3 mm^3^ into WNT974 (5 mg/kg daily oral gavage ×15), αPD1 (250 µg i.p. every 3 days ×4), WNT974+αPD1 (both) and control (methylcellulose + rat IgG at matching doses and intervals), followed by time-matched histological analyses. (*B*) IHC (n = 5 to 7 per group) was performed for Wnt7b protein in a time-matched manner post treatment with WNT974, αPD1, and the combination. (*C*) Immuno-fluorescence was performed on paraffin embedded sections for β-catenin on the same tissues as (*B*). (*D* and *E*) β-catenin staining was quantified as β-catenin positive cells (nuclear and perinuclear) divided by the total number of tumor cells. DAPI shows the nuclear stain. Response 1) Tumors with decreased Wnt7b and β-catenin expression by WNT974+αPD1 as compared to the control. Response 2) Tumors with no alteration or increase of Wnt7b and β-catenin expression by WNT974+anti-PD-1. *P < 0.05, Student’s *t* test. ****P < 0.0001, One-Way ANOVA followed by a test for multiple comparisons of means. (Scale bar, 20 µm.)

We previously showed that 005GSC is an αPD1-resistant IDH wildtype murine GBM model (8). Additionally, similar to human GBM, 005GSC is rich in stem cells, invasive and resistant to temozolomide (35, 36). These combined features make 005GSC a compelling model to study the role of acquired resistance to αPD1 via Wnt7b. Indeed, our time-matched immunohistochemistry (IHC) and Western blot analysis of 005GSC implanted mouse GBM tissues showed that αPD1 treatment led to elevated Wnt7b and β-catenin (nuclear and perinuclear) levels (Fig. 2 *A*–*D* and *SI Appendix*, Fig. S5*A*).

### Wnt Inhibition Reduces Resistance to αPD1 and Delays Growth of 005GSC GBMs.

**Fig. 3.**
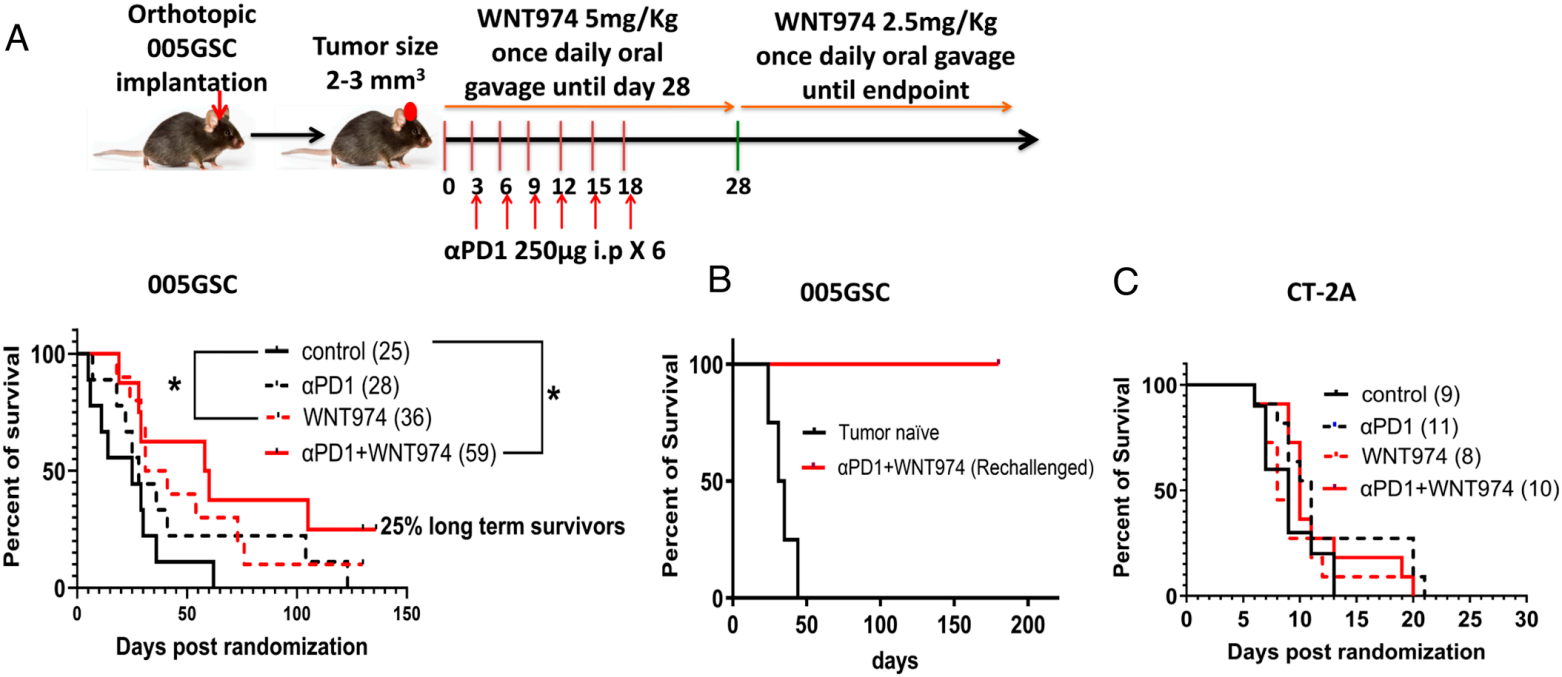
Wnt inhibition reduces resistance to αPD1 and improves survival of mice bearing 005GSC, but not CT-2A. (*A*) 30,000 parental 005GSC-GFP cells were orthotopically implanted and randomized into control, WNT974, αPD1, and WNT974+αPD1, and survival was monitored. n = 8 to 11 mice per group. The median survival for each arm is given in parentheses. *P < 0.05, Log-rank (Mantel-Cox) test. (*B*) 25% long-term survivors from experiments in (*A*) were rechallenged with 100,000 005GSC-GFP cells on their contralateral hemisphere. Percent survival is plotted, n = 2 to 4 mice per group. (*C*) 30,000 CT2A-GFP cells were orthotopically implanted and randomized into the same groups followed by monitoring of survival as in (*A*), n=8-11 mice per group.

To assess the causal role of Wnt-signaling in αPD1 therapy resistance, we utilized WNT974–an inhibitor of porcupine (PORCN), an ER-resident O-acyltransferase that mediates Wnt palmitoylation, an essential step in Wnt biosynthesis (41). First, we reproduced our published findings (4, 8) that 005GSC is resistant to αPD1 monotherapy (Fig. 3*A*). To investigate whether WNT974 in combination with αPD1 can improve the survival of tumor-bearing mice, we implanted 005GSC orthotopically, monitored tumor growth and randomized the mice into four groups–control (vehicle for WNT974+IgG for αPD1), WNT974, αPD1, and WNT974+αPD1. The median survival of the control group was 25 d. WNT974 improved the median survival significantly to 36 d, and WNT974+αPD1 increased the survival to 59 d (>twofold compared to control mice), with 2 out of 8 mice (25%) in the combination group surviving more than 135 d (Fig. 3*A*). Of interest, tumors did not form in the long-term survivors challenged with 005GSC cells in the contralateral hemisphere suggesting that these mice had developed a memory response and were cured (Fig. 3*B*). By contrast, WNT974 alone or in combination with αPD1 did not produce any survival benefit in CT-2A model (Fig. 3*C*). We observed that the lymphoid enhancer binding factor (LEF-1), a downstream protein in the Wnt pathway, was much higher in CT-2A as compared to 005GSC (*SI Appendix*, Fig. S3*C*). It is reasonable that tumors with LEF-1 activation independent of canonical Wnt ligand signaling, such as CT-2A, would not respond to WNT974. Indeed, WNT974 has been shown to be minimally effective in tumors with downstream activation of LEF-1: a mediator of Wnt signaling in EMT, brain metastasis, adenomatous polyposis coli, and β-catenin mutations (42, 43), since these tumors are not driven by aberrant Wnt ligand expression. The higher Wnt expression in 005GSC compared to CT-2A (*SI Appendix*, Fig. S3*C*) and higher downstream LEF-1 levels in CT-2A as compared to 005GSC suggest that CT-2A’s resistance to αPD1 is not related to the canonical Wnt pathway (8, 44). In fact, we have recently shown that antigen presentation deficiency and mesenchymal differentiation underlie resistance to immunotherapy in the murine syngeneic CT-2A tumor model (9).

In a third model, GL-261-MGH cell line, the mRNA expression of *Wnt* ligands was 200 to 500-fold lower than in 005GSC cell line; and Wnt7b protein level was ~10-fold lower in GL-261-MGH tumor tissue than in 005GSC tumor tissue (*SI Appendix*, Fig. S3 *B* and *C*). Interestingly, the combination of WNT974 and αPD1 did not prolong survival of GL-261-MGH bearing mice (*SI Appendix*, Fig. S3*D*). Collectively, our findings from the three αPD1-resistant models reveal a correlation between aberrant Wnt expression and response to WNT974 and αPD1.

Since WNT974 decreased the viable cells by 30% in vitro at 5 µM and 10 µM concentrations (*SI Appendix*, Fig. S4*A*), we used ApopTag to measure apoptosis in the WNT974, αPD1, and the combination groups but did not observe any difference as compared to control (*SI Appendix*, Fig. S4 *B* and *C*). Thus, WNT974 modestly decreases proliferation of 005GSC, but has no effect on apoptosis.

### Reduction in Wnt7b/β-Catenin in 005GSC Tumor Cells Improves Therapeutic Outcome via an Increase in Antigen Presentation and the Ratio of Proliferating CD8/Treg in the TME.

To dissect the mechanism of response, we interrogated whether WNT974 alone and/or in combination with αPD1 attenuated the Wnt pathway proteins and associated oncogenic signaling in tumor cells in vivo. We found that WNT974 monotherapy modestly decreased Wnt7b but did not affect β-catenin levels in the tumor tissues (Fig. 2 *B*–*E*). Intriguingly, WNT974 addition to αPD1 led to two strikingly different responses. In some tumors, WNT974+αPD1 attenuated endogenous- and αPD1-induced Wnt7b and β-catenin (Response 1). In others, WNT974+αPD1 did not change or increased Wnt7b and β-catenin levels as compared to the control (Response 2) (Fig. 2 *B*–*E* and *SI Appendix*, Fig. S5*A*). The tumor burden of the Response 1 group was significantly less than control tumors, whereas the tumor burden of the Response 2 group was similar or higher than the control tumors (*SI Appendix*, Fig S5*C*). By analyzing the phosphoproteome of the tumor tissues, we identified that pro-oncogenic pathway proteins such as mammalian target of rapamycin (mTOR) and mitogen-activated protein kinase/extracellular signal-regulated kinase (MAPK/ERK) were highly phosphorylated at p-mTOR^Ser2448^ and pP44/42^Thr202/Tyr204^ sites, respectively, particularly in Response 2 (*SI Appendix*, Fig. S5*D*). In the GL-261-MGH model that had a lower Wnt7b level than 005GSC (*SI Appendix*, Fig. S3*C*), we found that WNT974+αPD1 increased pAkt and pMAPK/ERK1/2 as compared to the control group (*SI Appendix*, Fig. S9*F*). Activation of these pathways has been implicated in activating β-catenin and downstream signaling. This is evident from the increased Lef-1 protein levels associated with Response 2 (*SI Appendix*, Fig. S5*D*) in 005GSC and GL-261-MGH (*SI Appendix*, Fig. S9*F*).

**Fig. 4.**
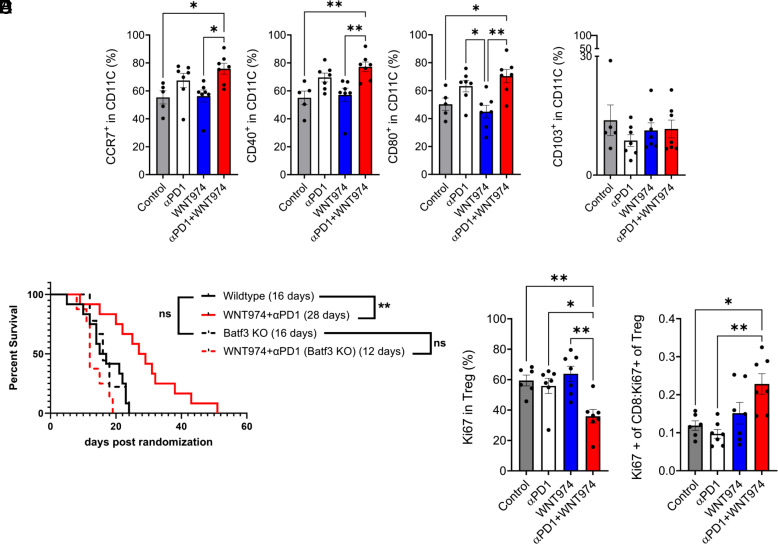
Response to therapy is due to increased antigen presentation and increased proliferative CD8/Treg ratio in the TME. (*A*) C57BL/6 mice were implanted with 30,000 005GSC followed by randomization at 2 to 3 mm^3^ into control, WNT974, αPD1 and WNT974+ αPD1. On day 15, posttreatment tumor was harvested, digested, passed through a mesh, stained with antibodies indicated, made into single cells, and subjected to flow analysis. CD45+CD11C+ cells were identified as dendritic-like cells. Out of the CD45+CD11C+%, the CD40%, CCR7%, CD80%, and (*B*) CD103% were analyzed. n = 5 to 7 mice per group. *P < 0.05, **P < 0.01. One-Way ANOVA followed by a test for multiple comparisons of means. (*C*) 30,000 005GSC were implanted in *Batf3^−/−^* mice with or without WNT974+ αPD1. The survival benefit observed in wild-type mice was lost in *Batf3^−/−^* mice. n = 8 to 13 mice per group, **P < 0.01 Log-rank (Mantel-Cox) test. In the experimental set up of (*A*), proliferative Treg cells % (*D*) and ratio of proliferative CD8: Treg cells (*E*) was determined. *P < 0.05, **P < 0.01, One-Way ANOVA followed by a test for multiple comparisons of means.

To elucidate the mechanisms that drive tumor cell death and elimination, we analyzed the tumor immune microenvironment (TIME) after treating mice with WNT974 and αPD1. We found that combined treatment with WNT974 and αPD1 maintained the overall hematopoietic immune cells marked by CD45. However, we detected an increase in the proportion of DC3-like dendritic cells (DCs), which were previously reported to express CCR7, CD80, CD40, or combination thereof (Fig. 4*A*) (45–48). DC3-like DCs share features with conventional dendritic cell subtypes. To ensure the DC phenotype we observed is related to function, we implanted 005GSCs in *Batf3*^−/−^mice that have reduced cDC1. The survival benefit observed in wild type mice by treatment with WNT974+αPD1 was abrogated in *Batf3*^−/−^ mice (Figs. 3*A* and 4*C*), suggesting that antigen presentation within the TME is a key driver of response. Whereas the CT-2A model, which did not respond to WNT974+αPD1, has multiple defects in antigen presentation machinery (9).

The CD4 T cell and CD8 T cell frequencies did not change with treatments (*SI Appendix*, Fig. S8 *A* and *B*). However, their cytotoxic activity was enhanced following combination therapy, as indicated by an increased proportion of Granzyme B (GzmB)+ CD8 T cells (*SI Appendix*, Fig. S8*D*). There were no significant differences in the levels of exhaustion markers (PD1, Lag3, Tim3) or the frequency of effector memory phenotype (CD127^hi^CD62L^lo^) (49) in CD8 T cells between the WNT974+αPD1 treated group and control group (*SI Appendix*, Fig. S8 *E* and *F*). Intriguingly, we observed an increase in % Treg cells (*SI Appendix*, Fig. S8*C*) which may be a potential resistance to WNT974+αPD1 treatment. However, these Treg cells were the least proliferative in the WNT974+αPD1 group (Fig. 4*D*); this may suggest that these Treg cells are less likely to exert immunosuppressive functions. Most importantly, the ratio of proliferative CD8 T cells to that of proliferative Treg cells increased twofold as compared to control and αPD1 monotherapy groups (Fig. 4*E*), indicating that the combination treatment alleviated resistance mediated by αPD1 and remodeled the immune suppressive TIME to be more immune stimulatory and antitumorigenic.

### Reprogramming of MDSCs Is Implicated in Resistance to WNT974+ αPD1.

**Fig. 5.**
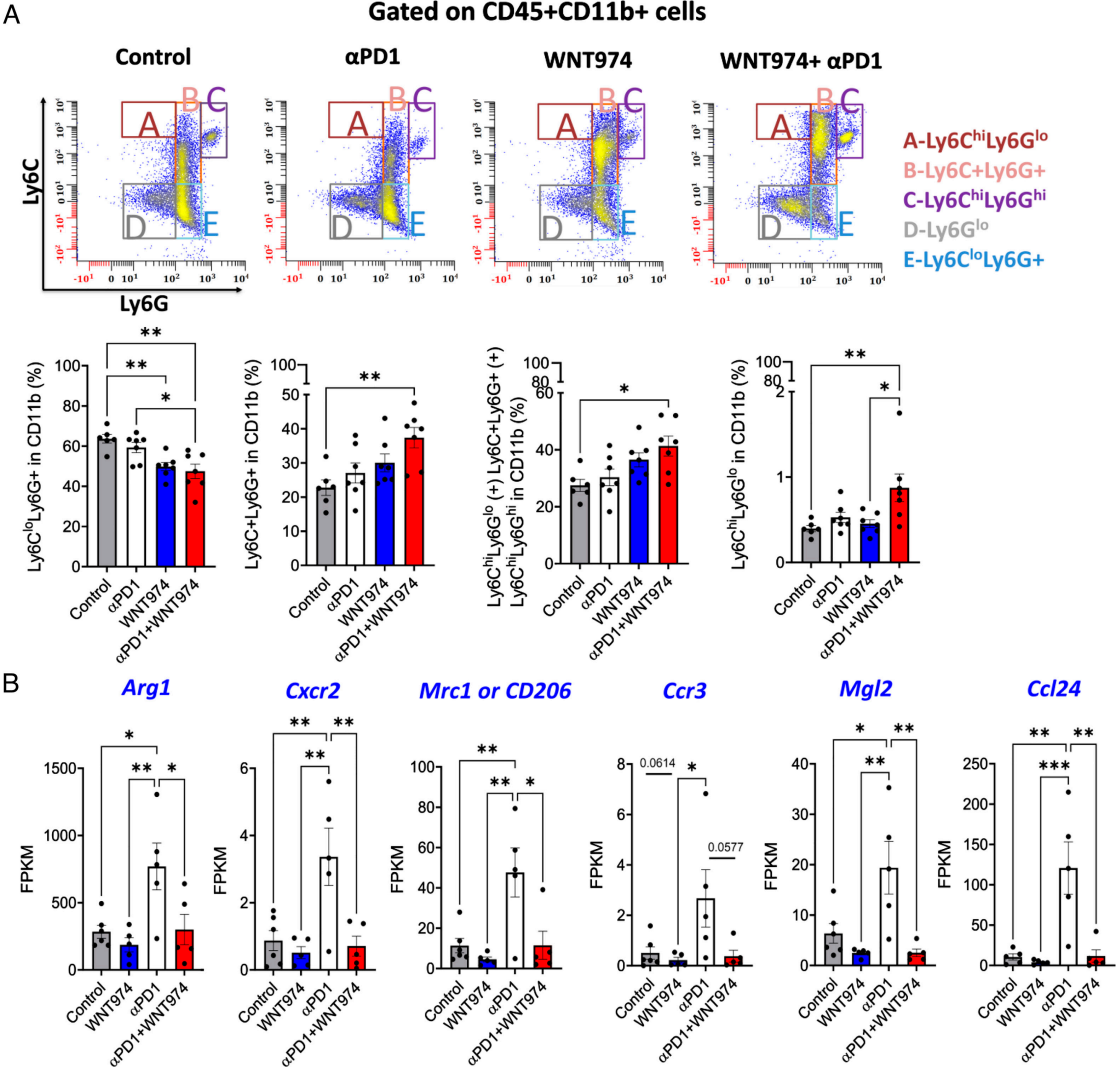
Reprogramming of MDSCs is implicated in resistance to WNT974+αPD1. (*A*) CD45+CD11b+ cells were gated for Ly6G and Ly6C populations. Ly6C^lo^Ly6G+% were identified as gMDSCs; Ly6C+Ly6G+ double positive populations were also analyzed. CD45+CD11b+Ly6C+ (includes Ly6C^hi^Ly6G^lo^, Ly6C+Ly6G+, and Ly6C^hi^Ly6G^hi^ cells) and CD45+CD11b+Ly6C^hi^Ly6G^lo^ mMDSC populations were gated and graphed. Winlist was used to show the gating strategy in a representative bivariant plot. (*B*) Bulk RNA-seq showing the Fragments per kilobase of transcript per million mapped reads of *Arginase1*, *Cxcr2, Mrc1, Mgl2,* and *Ccl24* genes. n = 5 to 7 mice per group, *P < 0.05, **P < 0.01, ***P < 0.001, One-Way ANOVA followed by Tukey’s post hoc test for multiple comparison of means.

Beyond DCs, we examined various subpopulations of CD45+ cells and found that Iba1+ cells, CD45+CD11b+ myeloid cells, CD45+CD11b+Ly6G-Ly6C-F4/80+ tumor-associated macrophages, CD45+CD11C+ or CD45+CD11C+CD103+ DCs were not altered by WNT974+αPD1 treatment (Fig. 4*B* and *SI Appendix*, Fig. S6 *A*–*C*). Within MDSCs, however, CD45+CD11b+Ly6C^lo^Ly6G+% gMDSCs decreased in the combination group as compared to control or αPD1 group (Fig. 5*A*). A double-positive population of Ly6C+Ly6G+ cells increased in the combination group as compared to the control group (Fig. 5*A*). By investigating the potential players in mediating the reduction in gMDSCs via bulk RNA-seq analysis of tumors, we found that αPD1 treatment a) increased the expression of *Arginase1, Mrc1 or CD206, and Mgl2*—genes associated with immune suppression by MDSCs and tumor-brain interfacial macrophages/microglia (50–52), b) increased *Ccl24* and c) led to a trend toward increased *Ccr3,* a receptor of *Ccl24* involved in eosinophil, T cell, and neutrophil chemotaxis (Fig. 5*B*). WNT974 addition to αPD1 alleviated these αPD1-induced changes in expression. Given the role of the vascular cell adhesion molecule 1 (VCAM1) in neutrophil infiltration (53–55), we performed a Western blot for VCAM1 in tumor tissue lysates (n = 3 mice per group). We found that WNT974 decreased VCAM1 levels to a greater extent than αPD1. The combination of WNT974 and αPD1 showed a reduction comparable to that of WNT974 as compared to control mice, indicating that blocking Wnt signaling is the main reason for the decrease in VCAM1 (*SI Appendix*, Fig. S7*A*). This pattern is consistent with the effect of WNT974, αPD1, and the combination on gMDSCs (Fig. 5*A*).

Our time-matched flow analysis indicated that the CD45+CD11b+Ly6C+ cells (that includes Ly6C^hi^Ly6G^lo^, Ly6C+Ly6G+, and Ly6C^hi^Ly6G^hi^ cells) increased in the WNT974+ αPD1 group as compared to the control (Fig. 5*A*). Further, after eliminating the Ly6G+ and Ly6G^hi^ cells, a purer monocyticMDSC (mMDSC) population CD45+CD11b+Ly6C^hi^Ly6G^lo^% increased in the WNT974+ αPD1 group as compared to the control or the WNT974 monotherapy group (Fig. 5*A*). A similar striking shift in the MDSC subpopulations was observed in the GL-261-MGH model with WNT974+αPD1 compared to the control (*SI Appendix*, Fig. S9 *A*–*E*). Specifically, there was a significant decrease in the proportion of CD45^+^CD11b^+^Ly6C^lo^Ly6G^+^ gMDSCs (gMDSCs) (*SI Appendix*, Fig. S9*B*), accompanied by an increase in CD45^+^CD11b^+^Ly6C^+^ mMDSC populations (*SI Appendix*, Fig. S9 *C*–*E*).

## Discussion

Wnt is essential for neurogenesis and regulation of the oligodendrocyte lineage. It is expressed in oligodendrogliomas and chemotherapy-resistant GSC progenitor cells (21). This indicates that dysregulation of Wnt signaling in GBM is an early event in gliomagenesis but is maintained in the World Health Organization IDH-wild type tumors (56). We detected elevated Wnt7b protein levels in 100% of IDH-wild type human GBMs tested in the tissue array with 70 GBM tumors and in IDH-wild type murine GBM 005GSC. CRISPR/Cas9-mediated knockout of *Wnt7b* in 005GSC implanted in immune-competent mice prolonged survival. In fact, the combination of Wnt inhibition and αPD1/L-1 has been tested in multiple extracranial tumors (57). Moreover, Zhang et al. showed that the tankyrase inhibitor XAV939 augmented the efficacy of αPD1 in GL-261 grown subcutaneously, instead of orthotopically (58). Our lab (59) and others (60) have shown that cancer cells grown in an orthotopic environment are resistant to ICBs, whereas the same cells grown in the flank are responsive to ICBs. We have also summarized the misleading conclusions drawn from preclinical studies that do not use orthotopic models (61). The GBM TME is uniquely immunosuppressive, and thus, our mechanistic findings offer heretofore unknown insights over previous extra-CNS studies. Moreover, unlike a previous study focusing on cancer-intrinsic Wnt/β-catenin signaling as a resistance mechanism to immunotherapy in GBM (62), our data suggest that αPD1 treatment-driven acquired resistance is relevant in addition to intrinsic resistance in developing more effective immune therapies for GBM (63). This information would benefit both preclinical and ongoing clinical studies using αPD1.

One major reason for the failure of therapies in GBM is intratumoral heterogeneity. Spatial profiling reveals regional heterogeneity in protein expression between distinct areas of the same GBM biopsy (64). Intratumoral heterogeneity correlates strongly with the enrichment of stemness in multiple cancers, including GBM (39). Ineffective antitumor responses may result from high subclonality associated with an immunosuppressive TME (65). Furthermore, due to their plasticity, GBM may switch between cell states in response to therapy and escape treatment (31, 66, 67). The first interesting finding from our study is that WNT974 and αPD1 combination phosphorylated and activated pro-oncogenic pathways such as mTOR and MAPK/ERK in 005GSC, and Akt and MAPK/ERK in GL-261-MGH, which have been implicated in activation of β-catenin in a context-dependent manner (68, 69). MAPK pathway alterations have been observed in the majority of ICB-responding GBMs and in one ICB nonresponding GBM (70). The MAPK pathway may increase IL-6 mRNA and protein (71), which in turn, induces CCL2 to recruit mMDSCs. IL6 can also activate NFKappaB and STAT3 in infiltrating monocytes and GSCs via IL6R and cause immune suppression (72).

Second, WNT974 in combination with αPD1 improved the survival of αPD1-resistant, 005GSC (Wnt^hi^), whereas CT-2A (Wnt^lo^) did not respond to either treatment. Of interest, 25% of the 005GSC-bearing mice treated with WNT974+αPD1 were cured.

Third, we identified a subset of DC3-like state of dendritic cells (CCR7+, CD40+, and CD80+) that mediate response to WNT974+αPD1 treatment in GBM. CD80, CCR7, and CD40 have been implicated in dendritic cell activation and maturation. CCR7+ DCs can migrate to lymph nodes to activate naïve T cells (73, 74). Our observation that CD103 is not associated with the DC3-like dendritic cell state is in line with other reports (45–48).

Although it has been reported that human DC3s develop via a specific pathway activated by GM-CSF, independent of cDC restricted and monocyte-restricted progenitors, they share features with conventional human DC subtypes. For example, CCR7 and CD40 upregulation has also been linked to cDC1 and cDC2 states in human breast tumors (73). Our results in GBM showing the involvement of CCR7+ and CD40+ DCs and a lack of response to WNT974+αPD1 in *Batf3^−/−^* mice reveal the overlap between DC3-like cells and cDC1 (75).

Fourth, WNT974 and αPD1 is a promising therapeutic strategy that decreased the proliferation of Treg cells and increased the ratio of proliferative CD8 to proliferative Treg cells in the TME. In a set of 39 primary and recurrent GBM patients, Sayour et al. (76) discovered that while the absolute number of tumor infiltrating T cells may not be informative in predicting clinical outcome, the ratio of CD8+ T cells to Treg cells positively correlates with clinical outcome. Hence, it is likely that the WNT974 and αPD1 combination will improve clinical outcomes in GBM patients.

Fifth, unlike previous studies (57, 77), we observed a decrease in gMDSC-associated immune suppression and an increase in mMDSCs with WNT974 and αPD1 in two models-GL-261-MGH (lower stemness features) and 005GSC (stemness-rich) with different phenotypes. Notably, this suggests that the effects of WNT974+αPD1 on the myeloid compartment are not restricted to tumors with high Wnt7b expression or stem-like phenotypes but may instead represent a conserved immunomodulatory response across diverse GBM models. MDSCs consist of a heterogeneous population (78) and there are limited studies that define their role or target them in GBM (79, 80). Markers that distinguish their phenotype and function are limited. Published single cell studies have not revealed the full portrait of MDSCs (6, 7, 31) due in part to their lower number as compared to TAMs and to the functional and phenotypic similarity of gMDSCs to neutrophils. In our study, the basal % of the Ly6G and Ly6C MDSC populations match the % reported for 005GSC (79, 81) and have the potential to impact T cell activation and proliferation (82). Our results are in line with the findings that gMDSCs account for a significant fraction of MDSCs in the TME (83, 84). We used equal numbers of male and female mice in our MDSC profiling experiment and did not observe sexual dimorphism in MDSCs as previously reported (80).

Last, our finding that the combination of WNT974 and αPD1 decreases VCAM1 protein in GBM has not been reported before. Reduction in VCAM1 levels with WNT974 alone, and the combination may be correlated with the reduction in gMDSC infiltration (85, 86). However, it is possible that reduction in VCAM1 could also impede T cells from infiltrating into the tumor. Additionally, among the 49 candidate ligand–receptor interactions associated with mesenchymal transition by longitudinal analysis and single-cell RNA-seq (25) identified VCAM1 on differentiated-like GBM tumors and myeloid cell expressing ITGB7 and ITGB1 as ligand–receptor pairs. Therefore, our results on the decrease of VCAM1 in tumors are in line with the well-known role of Wnt in EMT.

Our study also has some limitations. i) Wnt inhibition may be overcome by activation of upstream genes, e.g., ASCL1 that are critical for the maintenance of GSCs (87) and other driver mutations. ii) The functionality of DCs and MDSCs identified in the study may need to be assessed using cross-presentation assays and T cell suppression assays, respectively, with WNT974 and αPD1 for which DCs and MDSCs could be isolated from lymph nodes and bone marrows respectively rather than the TME due to their low numbers in the TME. iii) Whether Wnt elevation in stem-cell rich GBMs is a specific pathway of resistance to αPD1 or for other immune therapies such as anti-CTLA-4 is an interesting area for investigation. iv) Especially, a direct link between Wnt7b and resistance to ICB should be further explored using models with *Wnt7b* overexpression or alternative syngeneic models that exhibit high endogenous *Wnt7b* expression, beyond the 005GSC model used in this study. Currently GBM patient-derived organoid models face significant constraints in studying ICB resistance mechanisms. Specifically, these models often lack autologous T cells, do not include functional blood–brain barrier elements, and fail to adequately replicate the intricate interactions between GBM cells and the extracellular matrix (88). Therefore, further methodological advancements are essential to enhance the translational relevance of these models for investigating ICB resistance mechanisms such as increased Wnt signaling. By contrast, longitudinal analyses, an emerging approach in GBM clinical trials (89), using pre- and post-ICB treatment samples from human GBM patients could reveal correlations between *WNT7B* expression and treatment response. In fact, a recent retrospective pancancer analysis by Wang et al. (90) demonstrated a negative correlation between *WNT7B* expression and the expression of 50 immune checkpoint genes across multiple tumor types, including glioma. Additionally, *WNT7B* expression was inversely associated with immune cell infiltration rate and tumor neoantigen production capability. These findings suggest that *WNT7B* may contribute to reduced responsiveness to ICB therapies, including αPD1 treatment.

**Fig. 6.**
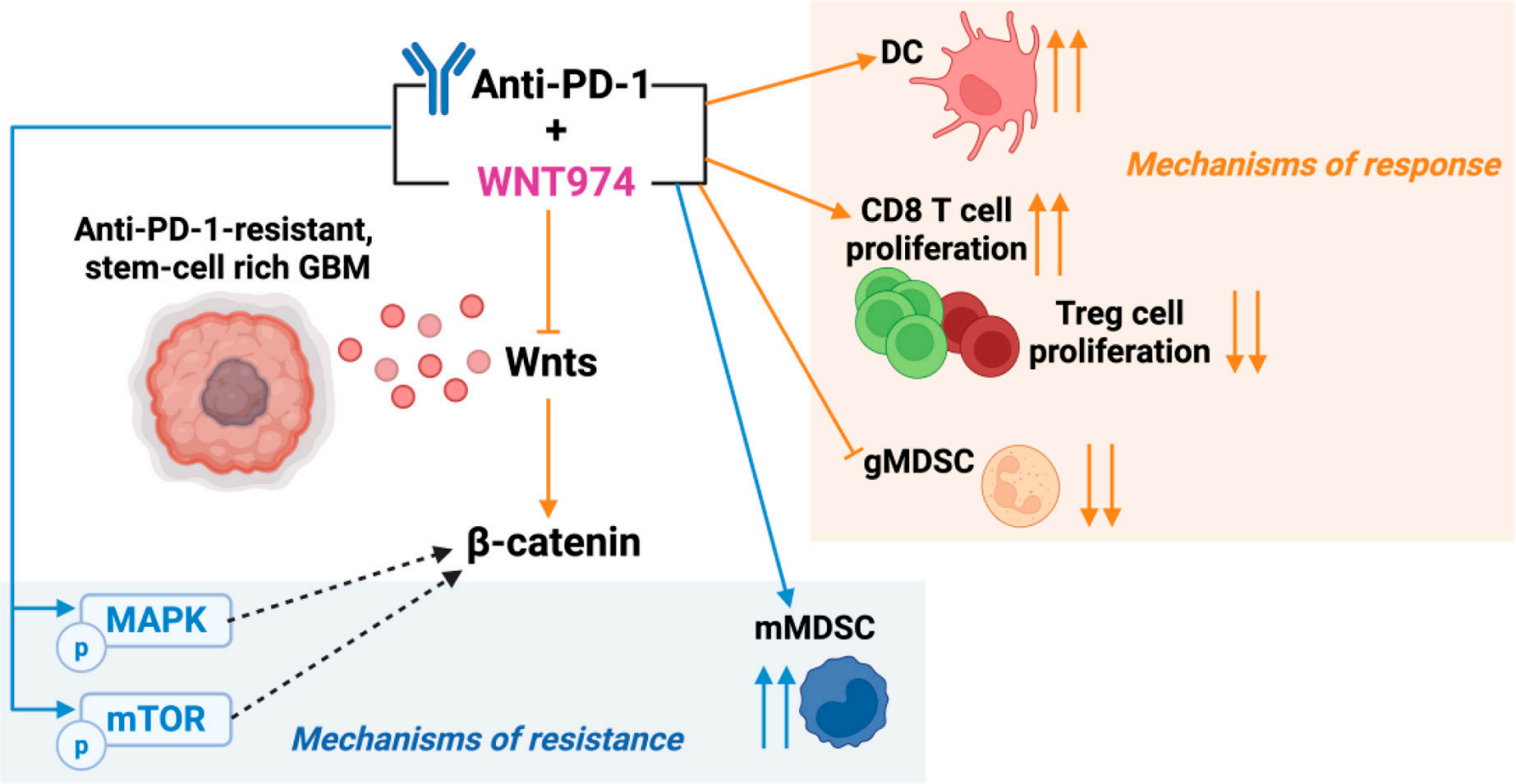
Mechanisms of response and resistance to Wnt inhibition and αPD1 in GBM. Response to WNT974 and αPD1 is likely mediated by an expansion of DC3-like DCs capable of antigen presentation, reduction in gMDSCs and associated immune suppression, and increased ratio of proliferative CD8/Treg. This is accompanied by an increase in mMDSCs as a mode of resistance. Resistance to WNT974 and αPD1 is also likely mediated by pro-oncogenic pathways such as mTOR and MAPK/ERK that could activate β-catenin and sustain downstream Wnt signaling. (*Figure created in BioRender*)

In a fatal disease like GBM where all phase III immunotherapy trials have failed (1, 3) and limited survival benefits noted with the current standard of care, we have identified the Wnt7b/β-catenin pathway as a potential biomarker of response to WNT974 and αPD1. This biomarker can guide clinical studies and help develop future preclinical models, enabling a better understanding of GBM. A phase I clinical trial of WNT974 in patients with advanced extracranial tumors showed that WNT974 was generally well tolerated (23, 91). By contrast, XAV939 used by other investigators (58) is highly toxic and is still in preclinical testing. Results from the phase I αPD1 antibody spartalizumab (PDR001)+WNT974 (NCT01351103) trial in non-CNS tumors show that such a combination increases T cell inflammation signature in some tumors, but decreases the signature in other tumors that were originally T cell inflamed, thus making them “cold” (42). The resistance mechanisms that we have identified (increased phosphorylation of protumorigenic pathway proteins, increased mMDSCs) may help explain the results of this extra-CNS tumor trial and inform the design of future GBM trials (Fig. 6).

## Materials and Methods

Cranial windows were implanted in C57BL/6 mice, followed by orthotopic glioma cell implantation using established protocols (8, 92, 93). Tumor growth was monitored using ultrasonography or blood Gluc, and mice received WNT974, αPD1, combination therapy, or control. Survival studies were done; and time-matched analyses included flow cytometry as previously described (92), Western blotting, immunohistochemistry, and bulk RNA sequencing of treated tumors. Human GBM tissues were sourced from clinical samples or tissue arrays; single-cell RNA-seq datasets were analyzed for Wnt7b expression in the GBM TME. Bulk RNA-seq data from TCGA were further analyzed for Wnt7b expression and survival correlations among GBM molecular subtypes, using appropriate statistical tests.

Please refer to the *SI Appendix* for a detailed description of the procedures and materials used.

## Supplementary Material

Appendix 01 (PDF)

## Data Availability

All study data are included in the article and/or *SI Appendix*.
