# Supplementary material for: Wnt inhibition alleviates resistance to anti-PD1 therapy and improves antitumor immunity in glioblastoma
Source: Proc Natl Acad Sci U S A. 2025 Sep 15;122(38):e2414941122. doi: 10.1073/pnas.2414941122 (PMC12478157; doi:10.1073/pnas.2414941122)
Supplement: Supplementary file 1 — Appendix 01 (PDF) [file pnas.2414941122.sapp.pdf]

Supporting Information

Supplementary Methods

Cell lines, cell culture, and animals:

CT-2A is a mouse astrocytoma that was formed by chemical induction by methylcholanthrene in C57BL/6 mice; p53 wild type, PTEN deficient). CT-2A was obtained from Dr. Thomas Seyfried of the Boston College. 005GSC-GFP was derived from GBM tumors induced by lentivirus transduction of H-Ras and Akt into the stem cell niche of *Tp53*$^{+/-}$ immunocompetent C57BL/6 mice. We received the 005GSC-GFP from Dr. Samuel Rabkins, MGH, Boston. GL-261 was provided by the Frederick National Laboratory, National Cancer Institute. MGG8 and MGG4, cell lines derived from GBM patients, were previously established in the Department of Neurosurgery at Massachusetts General Hospital (MGH) (1). All the cells were cultured in serum free NeuroCult NS-A proliferation kit (Stemcell Technologies) in a humidified atmosphere of 5% CO2 and 95% air at 37 °C. One day 10 post cranial window implantation, 30,000 cells (unless specified) in 1ul of PBS were stereotactically implanted 2 mm left of the sagittal suture, 0.1 mm rostral of the bregma, and at a depth of 2 mm from the brain surface. 6–8-week-old C57BL/6 mice were used for *in vivo* experiments. IACUC and the DoD review committee approved all animal procedures. All the murine cell lines used in this study are resistant to αPD1 in line with recent studies (2).

***In vivo* Treatment:**

6-8 weeks old C57BL/6 mice were implanted with 30,000 005GSC-GFP cells (unless specified), CT2A-GFP-Gluc or parental GL-261-MGH-Gluc. Mice were randomized at 2-3 mm^3 (unless specified) into control, WNT974, αPD1, and WNT974+αPD1. Mice were treated with 250ug αPD1or rat IgG i.p. once every 3 days for a total of 6 doses for survival studies and 4 doses for time-matched analyses. We had previously published that 5mg/kg of WNT974 once a day for 28 days was well tolerated and crossed the blood-brain barrier in mice (3). In the current work, WNT974 or equivalent methylcellulose was given by oral gavage at the dose of 5mg/kg for 28 days followed by 2.5 mg/kg for 28 days for survival studies and 5mg/kg once daily for 15 days for time-matched analyses.

Tumor growth measurement:

005GSC tumor growth was monitored through plastic cranial windows using a small animal ultrasonography device (Vevo 2100, FujiFilm VisualSonics Inc.) (4). The average of the longest and shortest diameters was taken for calculation of tumor volume. The growth of CT-2A-GFP-Gluc and GL-261-MGH was monitored by serial blood Gluc measurements using a Promega Glomax luminometer and correlating it with tumor volume as measured by ultrasound (5).

Flow cytometry

Tumors were micro-surgically isolated under a stereo-fluorescent microscope and weighed. Tumors were placed immediately in HBSS, and manual mincing produced a single-cell suspension. Samples were then individually filtered through 70-μm nylon strainers and incubated for 10 min on ice with rat anti-mouse CD16/CD32 mAb (clone 2.4G2/93) in PBS containing 2% BSA. After cell counting, samples were incubated for 45 min on ice in darkness with the following

conjugated antibodies from Biolegend at a dilution of 1:200: CD45 PerCP-Cy5.5 (clone 30-f11), CD103 PE-Cy7 (clone 2E7), CD11b BV785 (clone M1/70) CD11b PE-Dazzle (clone M1/70), CD11C-FITC (clone N418), F4/80 BV711 (clone BM8), CD19 APC (clone 1D3/CD19), CD80 BV421 (clone 16-10A1), CD40 PE-Cy5 (clone 3/23), CCR7 BV605 (clone 4B12), Ly6G FITC (clone 1A8), Ly6G-BV650 (clone 1A8), Ly6C PE-Cy7 (clone HK1.4), Ly6C APC (clone HK1.4), TCR PE-Dazzle (H57-597), TCR-beta BV711 (H57-597), CD3 BV570 (clone 17A2), CD4 BV650 (clone GK1.5), CD4 FITC (GK1.5), CD8-AF700 (clone 53-6.7), CD127 Pe-Cy7 (clone A7R34), NK1.1 PE-Dazzle (clone PK136), CD25 APC-Cy7 (clone PC61), CD127 BV605 (clone A7R34), CD62L BUV395 (clone MEL-14), PD-1 BUV737 (clone 29F.1A12), Tim3 PE-Fire810 (clone RMT3-23), Lag3 BUV805 (clone C9B7W), CD25 APC-Cy7 (clone PC61) and 1:100 of GZMB BV421 (clone GB11) and perforin PE (clone S16009A). Intracellular staining for ki-67 was performed after cellular permeabilization. Briefly, cells were fixed using a Foxp3/ Transcription Factor staining Buffer Set (eBioscience) for 15 min, followed by the addition of a permeabilization buffer. Cells were recovered by centrifugation. After washing steps with 2% BSA, the cells were filtered, and flow cytometry was performed on 1–1.5 million cells per individual sample on a BD LSRFortessa™ X-20. Intracellular staining was carried out using a primary antibody against Ki-67-APC (clone 16A8). All flow cytometric analyses were performed post hoc using FlowJo v10.1. Viable cells were identified based on side and forward scatter. Percentages were calculated over CD45+ cells or TCR+ cells.

Human Samples:

This study was approved by the Institutional Review Board at Brigham and Women's Hospital (BWH). Fifteen human GBM samples were identified through the surgical pathology files at the BWH. All of the patient samples were de-identified prior to the study. Patient samples were collected as part of Standard of Care (SoC) treatment. No informed consent was obtained since this was a retrospective study in which excess tissue was used from otherwise consented procedures as part of routine clinical care. Patient records were reviewed for age, sex, medical history, surgical intervention, therapeutic regimen, IDH1 mutation status, MGMT promoter methylation, and overall survival. A commercial human GBM tissue array containing 70 GBM patient samples and 10 normal brain tissue samples was purchased from US Biomax (GL805e).

Western Blot

Total cell lysate or nuclear-fractionated lysate was collected from cells/tissues in RIPA buffer supplemented with proteinase and phosphatase inhibitor cocktails, resolved by SDS-PAGE, and immunoblotted using standard techniques per the datasheet. 1: 1000 of Rabbit Wnt7b: Abcam #ab94915, 1:500 Rabbit Wnt5a Cell signaling #2392, 1:1000 of Rabbit Wnt7a: Abcam #ab100792, 1:1:1000 LEF-1: Cell signaling #2230, 1:1000 of Beta-catenin: Cell signaling #8480, 1: 1000 of mTOR (7C10): Cell signaling #2983S, 1:1000 of p-GSK alpha and beta: Cell signaling #8566S, 1:1000 of p-mTOR Ser 2448 Cell signaling #2971, 1:1000 of p44-42 MAPK (ERK1/2) (137F5) Thr202/Tyr204 Cell signaling #4695, 1:1000 of p-p44-42 MAPK (ERK1/2) Thr202/Tyr204 Cell signaling #4370S, 1: 1000 of Rabbit Wnt7b: ProSci #16-831, 1:1000 of Rabbit pLRP6 (Ser 1490) #2568, 1:1000 of Rabbit Cyclin D1: Cell signaling #55506, 1:1000 of p-Akt-Ser473 Cell signaling #4060, 1:1000 of Akt (11E7) Cell signaling #4685, 1:1000 of GAPDH Cell signaling #5174 and 1:5000 of mouse Beta-actin: Sigma, #A5441.

Quantitative Real-time PCR

For qRT-PCR the protocol from (3) was followed. Cells were lysed using RLT buffer. RNA was cleaned up with an RNeasy Kit (Qiagen) and DNAse-digested to remove DNA contamination. 100–500 ng of purified RNA was retro-transcribed using the High-Capacity RNA to cDNA Master Mix. qPCR was performed using SYBR Green master mix in Light-cycler 480 with specific primers designed for the Wnt pathway. *GADPH* was used as a housekeeping control. Reference genes and experiments were performed in duplicates for each sample.

Generation of *Wnt7b*$^{-/-}$ Cells

Two *Wnt7b* CRIPSR gRNA constructs, (gRNA 1 and gRNA 6, targeting exons 1 and 6) were purchased from (Gen Script:https://www.genscript.com/gRNA-detail/mouse/22422/Cas9/Wnt7b-CRISPR-guide-RNA.html#grna). The empty backbone vector (eSpCas9-LentiCRISPR v2) was used for control cells. Lentiviral particles were produced in-house following standard lab protocols. These particles were used to transduce 005GSC neurospheres. Virally transduced 005GSC cells underwent puromycin selection (0.5 µg/ml). Resistant cells were plated at low density by serial dilution in order to derive colonies originating from single cells. Individual colonies were assayed for Wnt7b protein levels and clonogenicity.

IHC & IF

Whole mouse brains were collected and fixed with 4% formaldehyde in PBS for 24 h. We next embedded the brains in paraffin, and 5-µm-thick paraffin sections were cut using a microtome and mounted on glass slides. 15 human GBM tissue sections were obtained from BWH. Commercial human GBM tissue array containing 70 GBM patient samples and 10 normal brain tissue samples was purchased from US Biomax (GL805e). Haematoxylin–eosin (H&E), Wnt7b (1:125, Abcam, #ab94915), β-Catenin (1:50, Cell Signaling Technology, #9562), and Iba1 (1:500, Wako #19741), staining was then performed. Anti-mouse Wnt7b was detected by DAB oxidation (IHC). β-Catenin, Iba1, and Wnt7b were detected by a cyanine3 (Cy3) fluorescence-conjugated secondary anti-rabbit antibody (IF, 1:200). For apoptosis analysis, mouse brain sections were stained with ApopTag fluorescein (Sigma-Aldrich, #S7160). IF sections were counterstained with DAPI (1:1000). Antigen retrieval was done on pH 9 solution at 97 °C for 20 minutes. To prevent nonspecific staining, sections were incubated with 5% normal donkey serum in PBS before incubation with the respective primary and secondary antibodies.

Quantification of IF Images

Stained sections were imaged with X20 objective lens using AxioScan Z1 slide scanner (Zeiss). All slides in each experiment and quantification were taken in the same imaging profile. Quantification of WNT7B and β-catenin positive cells was done using open source software, QuPath (6) First, the region of interest (ROI) was set in each slide as a cell-dense region for the mouse whole brain tissue slide, and whole tissue region for human GBM tissue slide. Total cell detection in ROI was done in each slide based on DAPI staining, followed by detection of specific cells expressing Cy3 signal using the ‘single measurement classifier’ function in the software. Final quantification was calculated as the percentage of Cy3 signal-positive cells among the total cell number in ROI.

RNA-seq in murine samples

RNA-Seq was performed according to (4) at the Massachusetts Institute of Technology BioMicro Center. Tumor samples were harvested at day 12 post-treatment initiation. The quality of RNA samples was confirmed using a fragment analyser (Advanced Analytical Technologies, Inc.) before Illumina libraries were prepared and indexed from ~250 ng of total RNA using the Kapa Hyperprep kit following the manufacturer's recommendations (Roche). Libraries were confirmed using the fragment analyzer and quantified by qPCR before pooling and sequencing on an Illumina NextSeq500 using 40 + 40 paired end reads. Samples were demultiplexed using custom scripts allowing a single mismatch to the index sequencing.

Bulk RNA-seq Analysis of GBM Subtypes

RNA-seq expression data for glioblastoma (GBM) patients were downloaded from The Cancer Genome Atlas (TCGA) via the GDC data portal in the form of transcript-per-million (TPM) normalized counts. Molecular subtypes (Classical [CL], Mesenchymal [MS], and Proneural [PN]) were annotated based on the GBM subtype classification framework from Wang et al. (7). For comparative gene expression analysis, we used log2(TPM + 1) transformed values and performed pairwise Wilcoxon rank-sum tests between subtypes. Resulting *p*-values were adjusted using the Benjamini-Hochberg (BH) method for multiple hypothesis correction.

Correlation and Survival Analyses

For survival analysis, patients were stratified into *WNT7B*-high and *WNT7B*-low groups based on median expression. Kaplan–Meier survival curves were generated using the survfit function from the **survival** R package, and log-rank tests were used to determine statistical significance (*p*-values) within each subtype cohort.

scRNA-seq Data Integration and Cell Type Annotation

Single-cell RNA sequencing datasets from multiple publicly available GBM cohorts ((8), GSE147275 (9), GSE154795 (10), GSE162631 (11), GSE163108 (12), GSE163120 (13)) with 123 patients were aggregated and integrated using the **Harmony** batch correction algorithm. Preprocessing steps, including normalization, variable gene selection, scaling, and principal component analysis (PCA), were performed using **Seurat v4**. UMAP dimensional reduction was applied to visualize cell clusters (14).

Cell type annotation was based on canonical marker genes and cluster-specific gene expression identified using the FindAllMarkers function (min.pct = 0.05, logfc.threshold = 0.1, test.use = "wilcox"). Marker genes for immune and tumor populations included *CD14, LYZ, TREM2, CD163, GFAP, SOX2*, and *SOX9*, among others. Tumor cells were defined based on high expression of *SOX2*, *SOX9*, *GFAP*, and lack of myeloid markers.

Macrophage Polarization and *WNT7B* Expression

Macrophage polarization was assessed by calculating M1 and M2 module scores using the AddModuleScore function in Seurat. M1 gene set included *IL1B*, *TNF*, *IRF1*, *S100A8*, and *S100A9*; M2 gene set included *CD163*, *MSR1*, *TREM2*, *IL10*, *MERTK*, and *ARG1*. For each patient, average module scores were computed separately for M1- and M2-like macrophages by grouping cells using group_by(sampleID, polarization_status) and summarizing scores accordingly. *WNT7B* expression was analyzed in tumor cells and stratified by M1/M2 ratio per patient to explore the relationship between macrophage polarization and tumor-intrinsic *WNT7B* expression.

Statistical Analysis:

Statistical analyses were performed using Graph Pad Prism Version 9.3.0. One-way ANOVA followed by Tukey's multiple comparisons test was performed for all experiments except for survival studies, where Log Rank test was used. $p < 0.05$ was considered significant. Two-tailed Unpaired Student's T test was used for comparison between the two groups. The number of samples or animals' 'n' is given in the Figure legends.

Supporting Information

Supplementary Figure 1

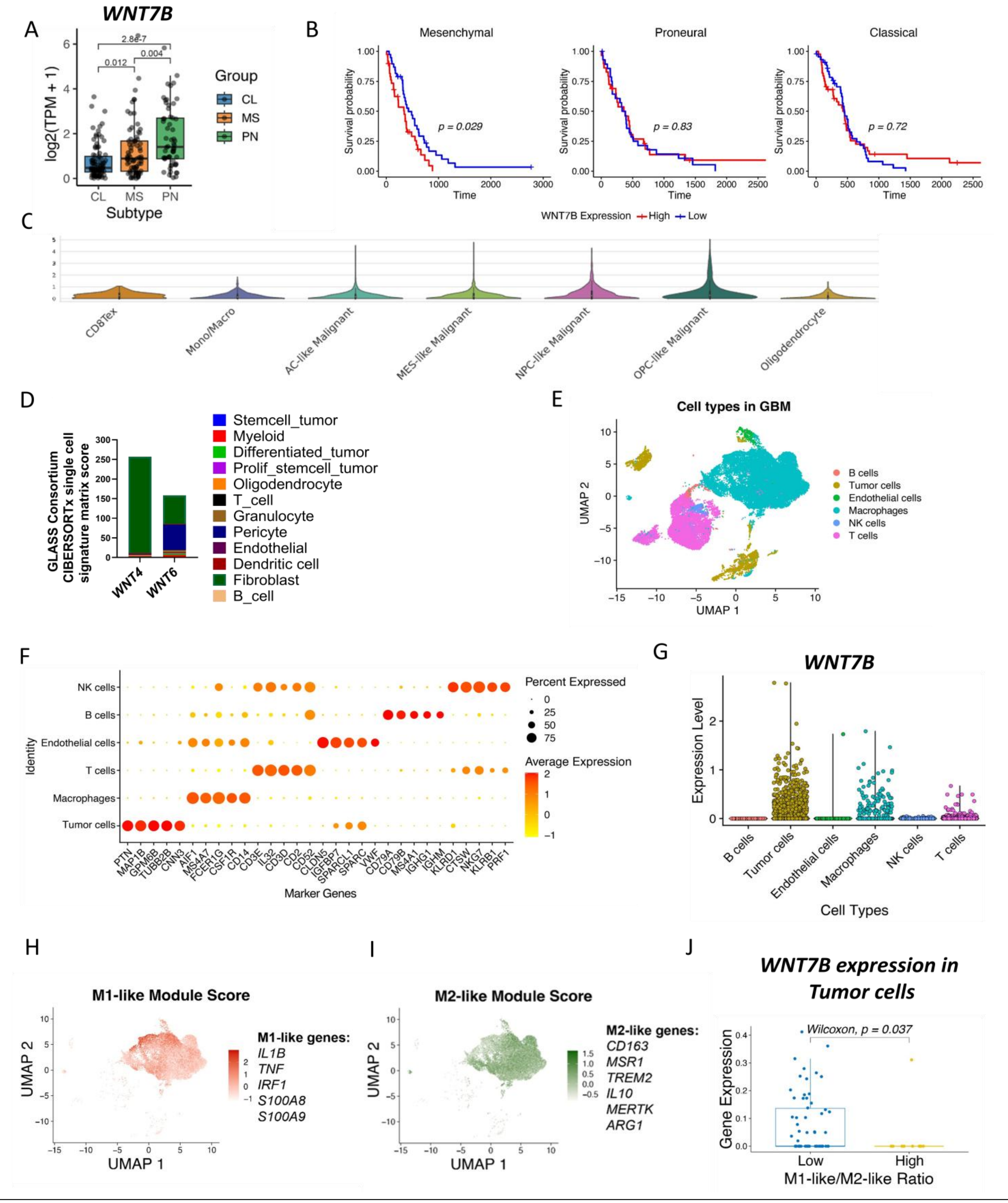

Fig. S1: Human GSC subtypes and the GBM TME populations that express *WNT7B*, and other *WNTs*

A) Boxplots of *WNT7B* expression across classical (CL), mesenchymal (MS), and Proneural (PN) GBM subtypes (*p*-values from Wilcoxon tests with BH correction). B) Kaplan–Meier survival curves of GBM patients stratified by high (red) vs. low (blue) *WNT7B* expression within mesenchymal, Proneural, and classical subtypes. High *WNT7B* expression is associated with reduced survival specifically in the mesenchymal subtype ($p = 0.029$). C) Violin plot of single cell RNA-seq of 28 GBM patient tumors from GBM_GSE131928_Smartseq2 dataset (8) (http://tisch.comp-genomics.org/home/) showing the expression *of WNT7B* in the tumor and its microenvironment. D) Deconvolution of the GLASS gene expression dataset of 168 GBM patient tumors (15) by applying CIBERSORTx (16) using reference cell-state signatures derived from 55, 284 single-transcriptomes from 11 adult patients spanning glioma subtypes and time points (17) shows that *WNT4* and *WNT6* are predominantly expressed in fibroblasts and pericytes. E) UMAP projection showing annotated cell types across integrated GBM single-cell RNA-seq datasets. F) Dot plot depicting expression of selected marker genes across identified cell types; dot size represents the proportion of expressing cells, and color indicates average expression. G) *WNT7B* expression across cell types, showing enrichment in tumor cells. Module scores for M1-like (H) and M2-like (I) macrophage gene programs across the UMAP. J) Average *WNT7B* expression in tumor cells stratified by patient-level M1-like/M2-like macrophage ratio.

Supplementary Figure 2

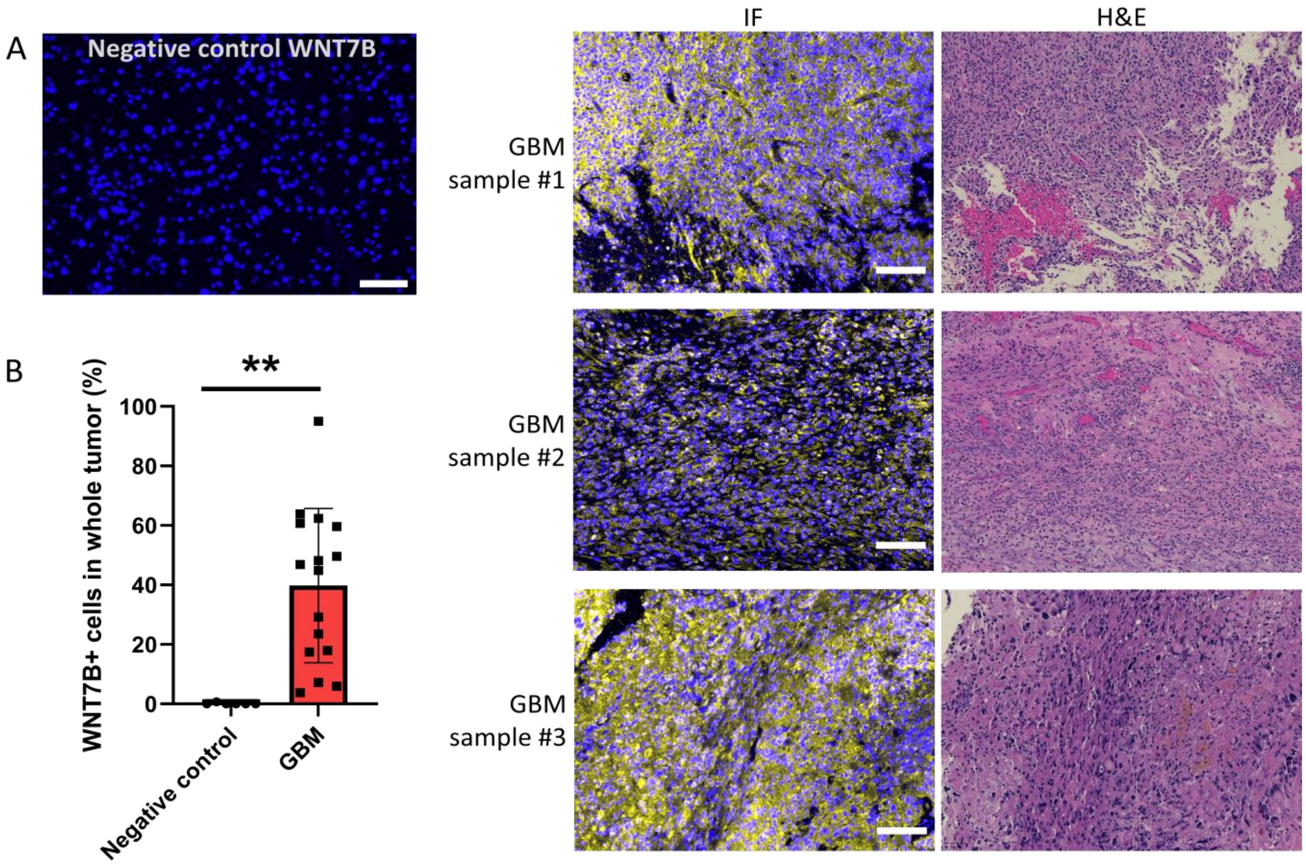

Fig. S2: WNT7B is highly expressed in human GBM patient tumor samples

A) Negative control is without a primary antibody but with Cy3-conjugated secondary antibody staining. Scale bar, 100 µM. B) GBM patient samples were stained for WNT7B protein using IF. Images of 3 representative patients from a total of 15 patients is shown. Adjacent sections stained with H&E are shown next to the IF images. DAPI stain is shown in blue. Secondary Cy3 stain is shown in yellow (saturated signal in white indicates high WNT7B expression). ** p-value < 0.01, Two-tailed, Unpaired student's t-test.

Supplementary Figure 3

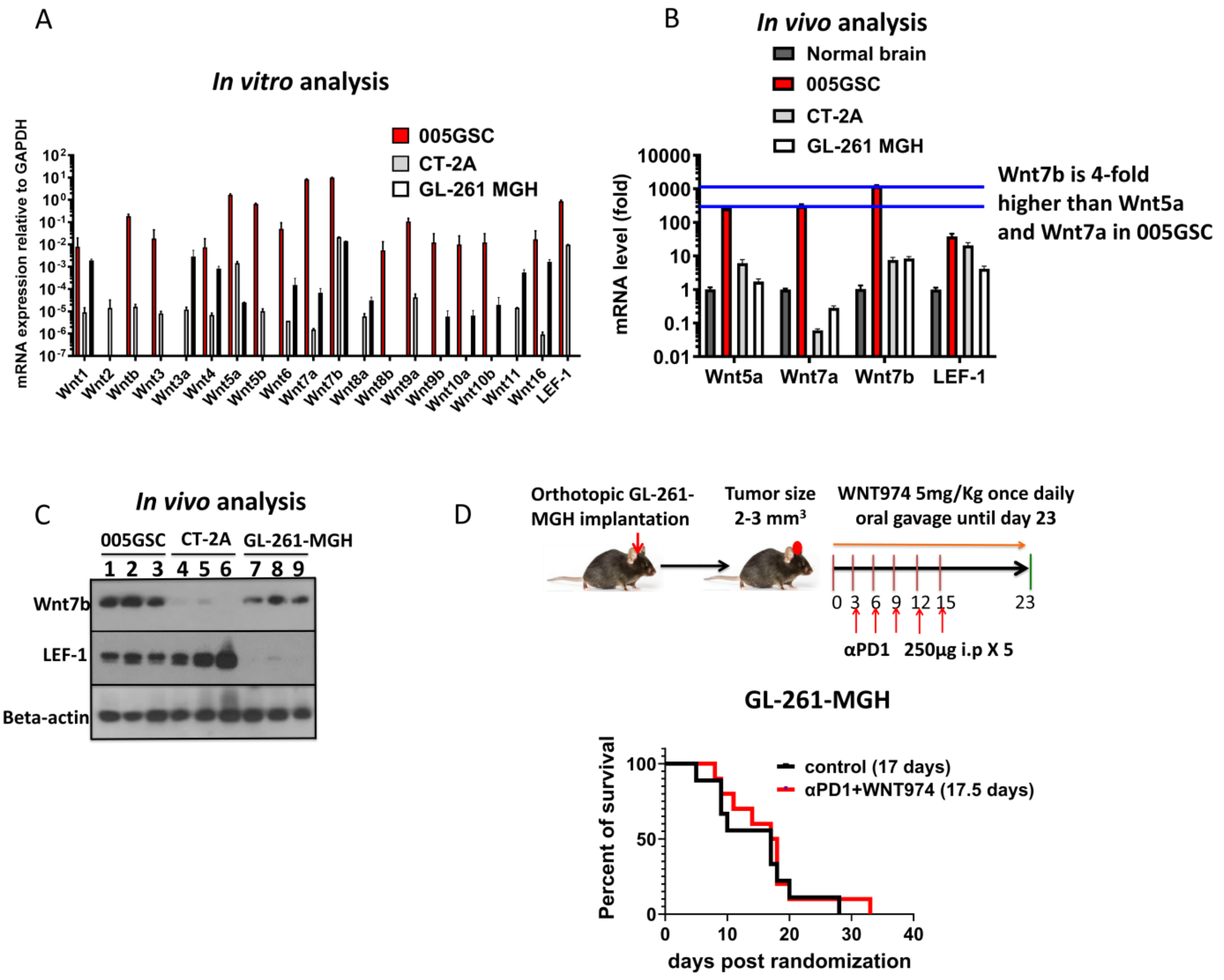

Fig S3: Elevated *Wnt* expression in 005GSC.

A) *In vitro* screening for the expression of 19 Wnt ligands relative to GAPDH in 005GSC, CT-2A and GL-261-MGH. B) *In vivo* mRNA expression of *Wnt5a*, *Wnt7a*, *Wnt7b* in normal brain, 005GSC, CT-2A and GL-261-MGH. The y-axis is in log scale. C) *In vivo* protein levels of Wnt7b and Lef-1 in tissue lysates of 005GSC, CT-2A and GL-261-MGH (n=3 samples per group). Beta-actin was used as a loading control. D) 30,000 parental GL-261-MGH cells were orthotopically implanted and randomized into control and WNT974+αPD1 groups; treatment schedule and dosing are shown; survival was monitored. n=10-11 mice per group.

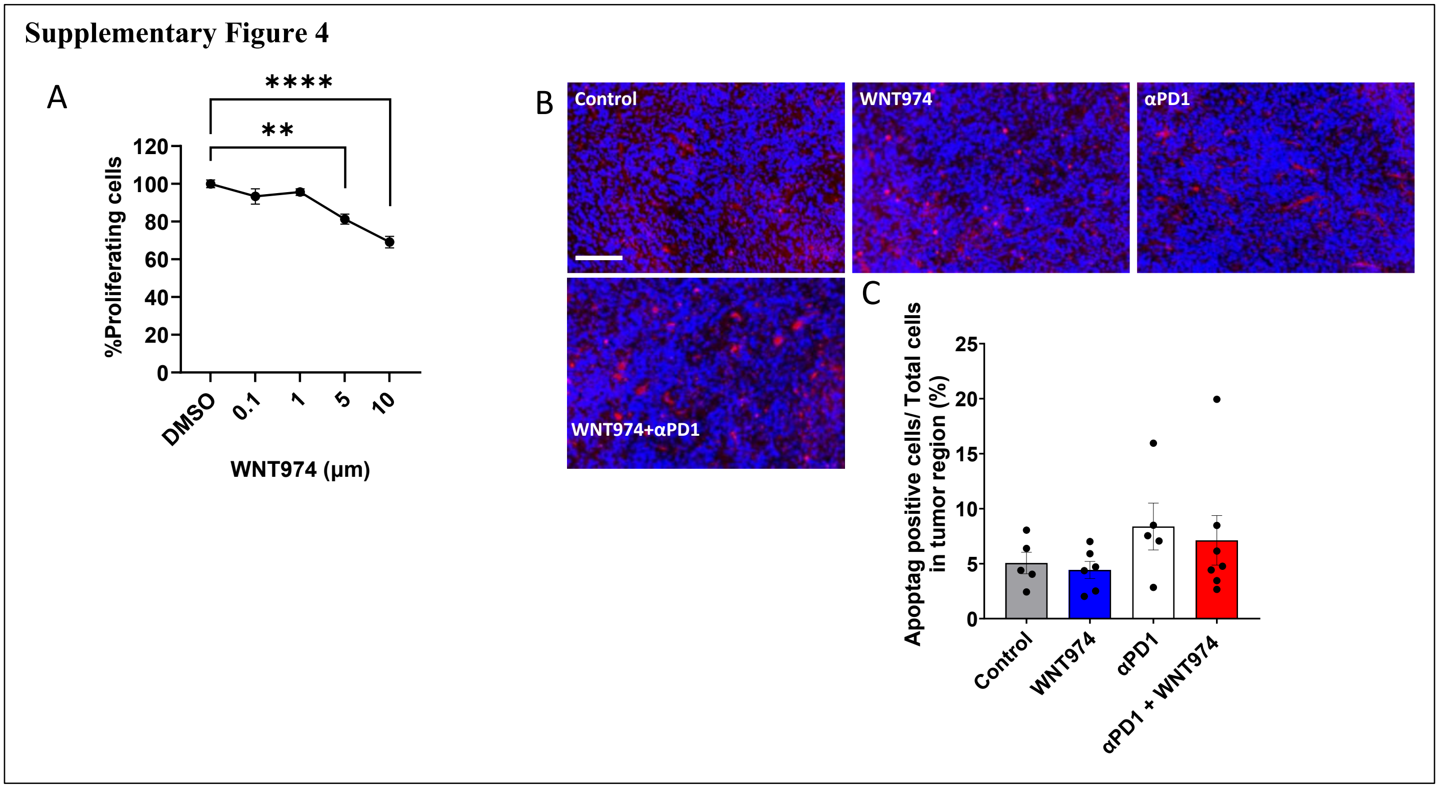

Fig. S4: WNT974 modestly reduces proliferation of 005GSC at higher concentrations.

A) MTT assay was performed to measure % viable 005GSC cells in increasing concentrations of WNT974 at 72 h post-treatment. B-C) For apoptosis analysis using WNT974, αPD1 or the combination, mouse brains were harvested in a time-matched manner, embedded in paraffin, sectioned, and stained with ApopTag fluorescein (Sigma-Aldrich, #S7160). Sections were counterstained with DAPI (1:1000). ** p-value<0.01, **** p-value<0.0001

Supplementary Figure 5

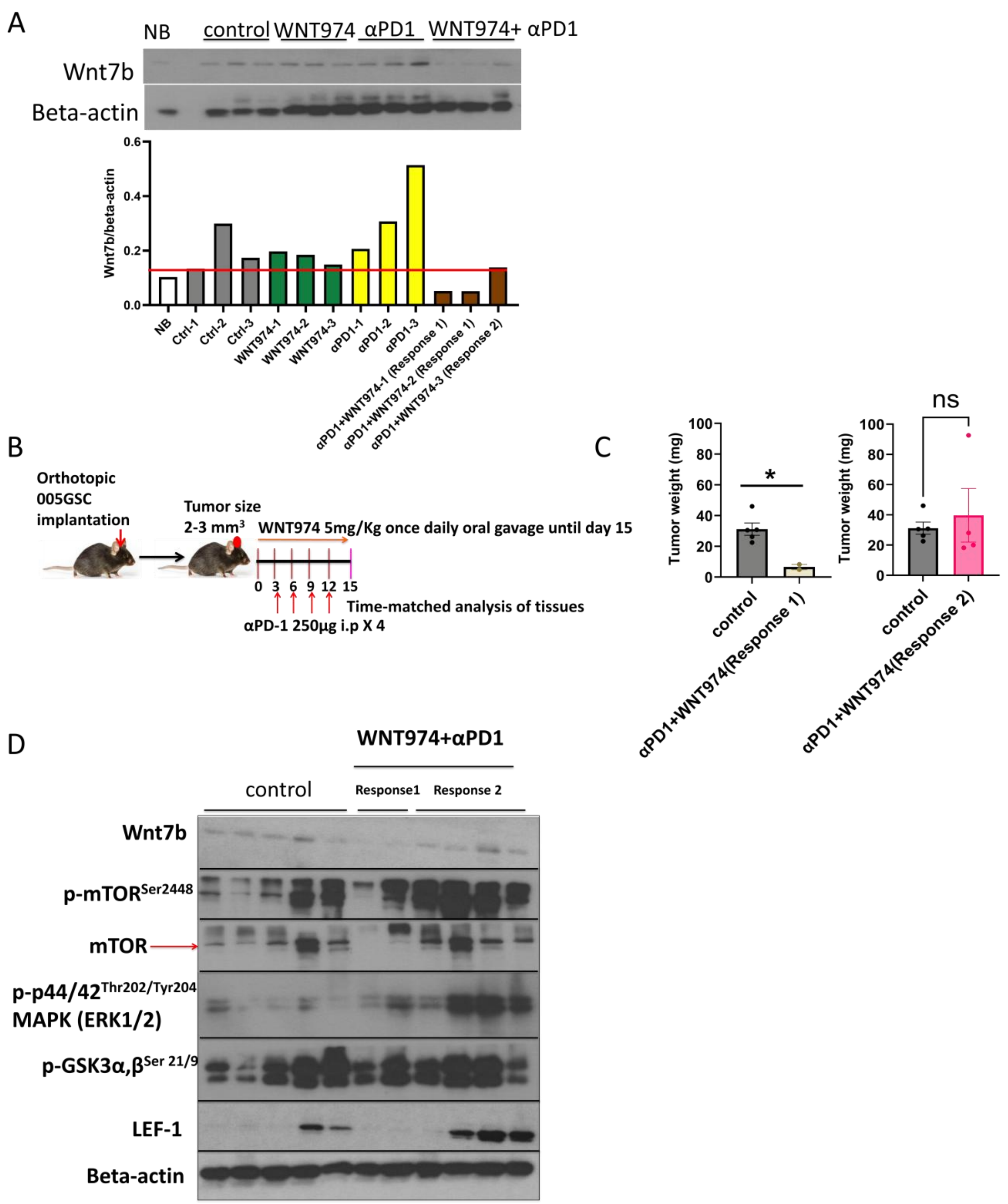

Fig. S5: Response to therapy is partly due to reduced levels of Wnt7b/β-catenin in 005GSC tumor cells.

A) C57BL/6 mice were orthotopically implanted with 30,000 005-GSC-GFP. Mice were randomized at 2-3 mm^3 into control, WNT974, αPD1, and WNT974+αPD1. Mice (n=3 per group) were treated with 250ug αPD1 or rat IgG i.p. once every 3 days for a total of 4 doses, WNT974 or equivalent methylcellulose was given by oral gavage at the dose of 5mg/kg once daily for 15 days. A time-matched Western blot was performed to determine Wnt7b protein levels. Normal brain tissue was used as a negative control. Beta-actin was used as a loading control. Densitometry shows individual tumor Wnt7b/beta-actin values. The red line

indicates the control group's sample with the lowest Wnt7b/beta-actin ratio; this was used as the baseline to distinguish response 1 and 2 to the tumors in the WNT974 + αPD1 group. Response 2 has Wnt7b level that is the same as the control whereas Response 1 has potently reduced Wnt7b as compared to the control B) Experiment A) was performed the 2nd time with the control and WNT974+αPD1 groups with (n=6 or 5) mice per group to analyse the proteome using Western blot. The experimental set up is shown. C) Tumor weight in mg at the time of harvest (day 15) of control mice as compared to Response 1 mice and Response 2 mice is shown. D) Western blot was performed on tumor tissue lysates treated with WNT974+αPD1 or control on day 15 with antibodies as indicated. *p value<0.05, Two-tailed, Unpaired student's t-test.

Supplementary Figure 6

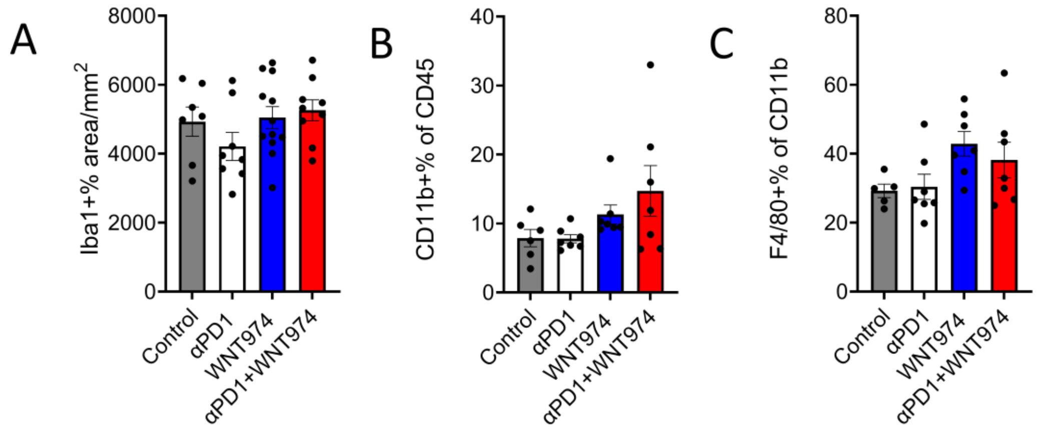

Fig. S6: WNT974 and αPD1 does not affect the microglial and macrophage frequencies

A) Immunofluorescence for Iba1 was performed on treated tissues in a time-matched manner. Iba1+ cells that include microglia, macrophages and monocytes was quantified per /mm^2 area, B) Flow cytometric analysis of treated time-matched tissues was performed and CD11b+ % in CD45% (myeloid cells) and C) F4/80+ % in CD45+CD11b+% (macrophage-like cells) was graphed. n=5-7 mice per treatment group.

Supplementary Figure 7

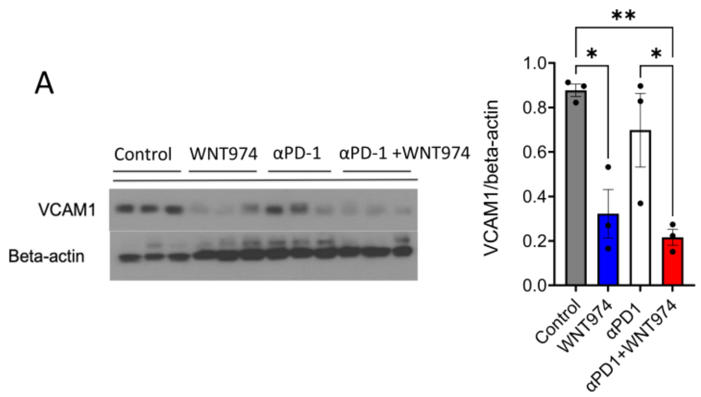

B

Candidate receptor-ligand interactions associated with mesenchymal transition

	Longitudinal analysis		
	**VCAM1 correlation against**	**p-value of VCAM-1**	**FDR**
ITGB7	R=0.325901578	0.000174028	0.000329509
ITGB1	R=0.667537075	7.64E-18	1.08E-16
	Single-cell RNA-seq analysis		
	**VCAM1 correlation against**		
ITGB7	R=0.435218303		
ITGB1	R=0.435218303		

Fig. S7: WNT974 and αPD1 reduce VCAM1 protein level.

A) Western blot showing the VCAM1 protein levels in treated time-matched tissues (n=3 per group) and its quantification with the treatment arms mentioned. Beta-actin is used as a loading control * $p<0.05$, ** $p<0.01$, ***$p<0.001$. One-Way ANOVA followed by multiple means comparison test. B) Out of the 49 putative ligand-receptor interactions, p values, FDR and Pearson correlations coefficients of the ligand VCAM1 for receptors ITGB7 and ITB1 obtained from longitudinal GBM patient biopsies and single cell sequencing analysis are indicated. *p value<0.05, **$p<0.01$, One-way ANOVA followed by Tukey's post-hoc test for multiple comparisons of means.

Supplementary Figure 8

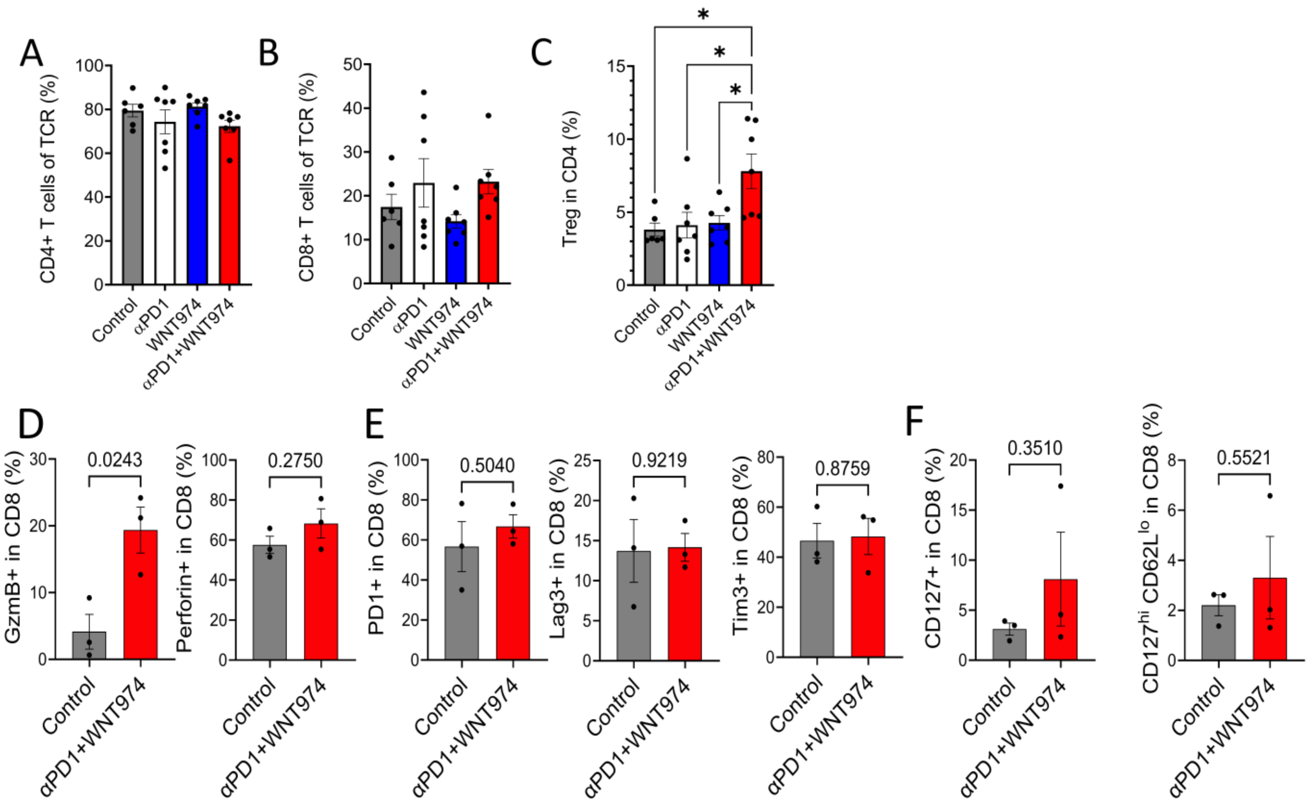

Fig. S8: WNT974 + αPD1 does not affect the % of CD4+ or CD8+ T cells, but affects the Treg cell and cytotoxic CD8+ T cell frequencies. Flow cytometric analysis of treated time-matched 005GSC tumor tissues was performed. A-B) CD4+ (%) and CD8+ (%) of TCR+ cells, and C) Treg cells (CD25+CD127^{lo}) of CD4 T cells were plotted. n=5-7 for each treatment condition. *p-value<0.05, One-way ANOVA followed by Tukey's post-hoc test for multiple comparisons of means. For analysis on specific phenotype or activated state of T cells, D) GzmB+ or Perforin+ cytotoxic CD8+ T cell, E) PD1+, Lag3+, or Tim3+ exhausted CD8+ T cell, F) CD127+ or CD127^{hi}CD62^{lo} effector memory CD8+ T cells were plotted between control and WNT974+αPD1 treated group. n=3 for each group. p-value<0.05 is significant, Two-tailed, Unpaired student's t-test.

Supplementary Figure 9

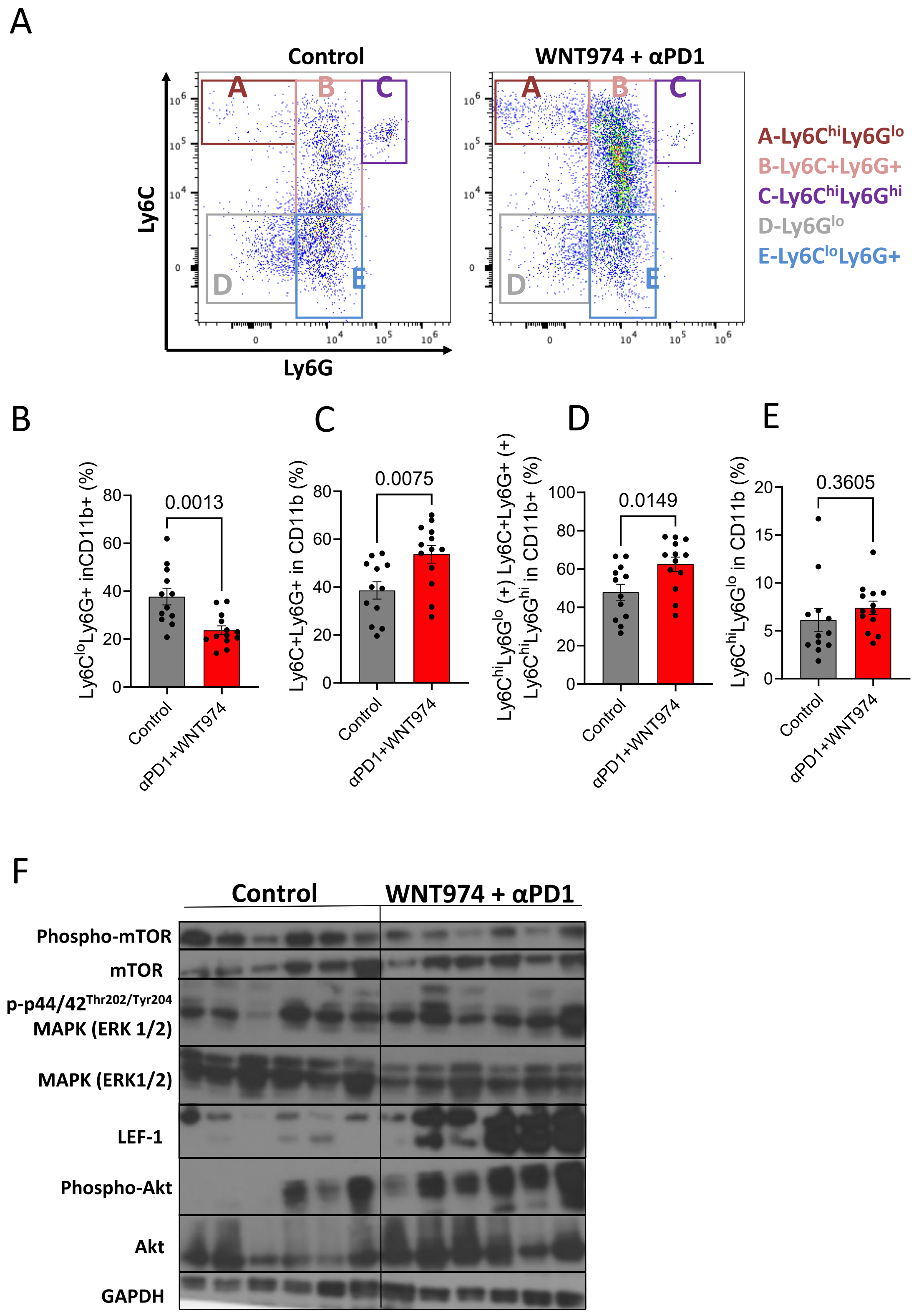

Fig. S9: WNT974 and αPD1 reprograms MDSCs and activated pro-oncogenic pathways in GL-261-MGH, a GBM model with lower Wnt7b levels than 005GSC.

A) 100,000 GL-261-MGH cells were implanted in C57BL/6 mice and randomized on day 10. The treatment regimen was as follows: 5 mg/Kg WNT974 for 15 days and 250 µg αPD1 X 4 once every 3 days starting from day 3 after randomization. On day 16 post-randomization tumor tissues were analyzed by flow. CD45+CD11b+ cells were gated for Ly6G and Ly6C populations. B) $Ly6C^{lo}Ly6G$+% were identified as gMDSCs in GL-261-MGH tumor tissue. C-E) Ly6C+Ly6G+%, and Ly6C+ (includes $Ly6C^{hi}Ly6G^{lo}$, Ly6C+Ly6G+, and $Ly6C^{hi}Ly6G^{hi}$ cells), and $Ly6C^{hi}Ly6G^{lo}$ were identified as mMDSCs. n=12 for Control and n=13 for WNT974+αPD1 treated condition. p-value<0.05 is significant, Two-tailed, Unpaired student's t-test. F) Western blot was performed on tumor tissue lysates treated with WNT974+αPD1 (n=6) or vehicle + IgG (n=6) at the same time point as a) with antibodies as indicated.

Supplementary Table S1 (Patient demographics)

The clinical characteristics such as age, gender, IDH1 mutation status, MGMT promoter methylation status, treatment regimen (such as TMZ/Radiation, ICB, bevacizumab) of GBM patients in which WNT7B staining was performed.

**Patient and tumor characteristics (n=15)**			
Recurrent/residual	9 (60%)		
Newly diagnosed	6 (40%)		
Age at diagnosis (years)		Sex, no (%)	
Median	58	Male	7 (47%)
Range	34-85	Female	8 (53%)
IDH1 R132H mutation status, no (%)		MGMT promoter methylation status, no (%)	
No	14 (93%)	Methylated	6 (40%)
Yes	1 (7%)	Unmethylated	9 (60%)
Treatment		Survival (months)	
Adjuvant chemo	14 (93%)	Median	40
Adjuvant Radiation	14 (93%)	Range	3-64
Bevacizumab	7 (47%)		
ICB	4 (27%)		

References

1. H. Wakimoto *et al.*, Maintenance of primary tumor phenotype and genotype in glioblastoma stem cells. *Neuro Oncol* **14**, 132-144 (2012).
2. M. Datta *et al.*, Losartan controls immune checkpoint blocker-induced edema and improves survival in glioblastoma mouse models. *Proc Natl Acad Sci U S A* **120**, e2219199120 (2023).
3. A. Griveau *et al.*, A Glial Signature and Wnt7 Signaling Regulate Glioma-Vascular Interactions and Tumor Microenvironment. *Cancer Cell* **33**, 874-889 e877 (2018).
4. G. Seano *et al.*, Solid stress in brain tumours causes neuronal loss and neurological dysfunction and can be reversed by lithium. *Nat Biomed Eng* **3**, 230-245 (2019).
5. J. Kloepper *et al.*, Ang-2/VEGF bispecific antibody reprograms macrophages and resident microglia to anti-tumor phenotype and prolongs glioblastoma survival. *Proc Natl Acad Sci U S A* **113**, 4476-4481 (2016).
6. P. Bankhead *et al.*, QuPath: Open source software for digital pathology image analysis. *Scientific reports* **7**, 16878 (2017).
7. Q. Wang *et al.*, Tumor Evolution of Glioma-Intrinsic Gene Expression Subtypes Associates with Immunological Changes in the Microenvironment. *Cancer Cell* **32**, 42-56 e46 (2017).
8. C. Neftel *et al.*, An Integrative Model of Cellular States, Plasticity, and Genetics for Glioblastoma. *Cell* **178**, 835-849 e821 (2019).
9. H. Shaim *et al.*, Targeting the alphav integrin/TGF-beta axis improves natural killer cell function against glioblastoma stem cells. *J Clin Invest* **131** (2021).
10. A. H. Lee *et al.*, Neoadjuvant PD-1 blockade induces T cell and cDC1 activation but fails to overcome the immunosuppressive tumor associated macrophages in recurrent glioblastoma. *Nat Commun* **12**, 6938 (2021).
11. Y. Xie *et al.*, Key molecular alterations in endothelial cells in human glioblastoma uncovered through single-cell RNA sequencing. *JCI Insight* **6** (2021).
12. N. D. Mathewson *et al.*, Inhibitory CD161 receptor identified in glioma-infiltrating T cells by single-cell analysis. *Cell* **184**, 1281-1298 e1226 (2021).
13. A. R. Pombo Antunes *et al.*, Single-cell profiling of myeloid cells in glioblastoma across species and disease stage reveals macrophage competition and specialization. *Nat Neurosci* **24**, 595-610 (2021).
14. Y. Hao *et al.*, Dictionary learning for integrative, multimodal and scalable single-cell analysis. *Nat Biotechnol* **42**, 293-304 (2024).
15. F. S. Varn *et al.*, Glioma progression is shaped by genetic evolution and microenvironment interactions. *Cell* **185**, 2184-2199 e2116 (2022).
16. A. M. Newman *et al.*, Determining cell type abundance and expression from bulk tissues with digital cytometry. *Nat Biotechnol* **37**, 773-782 (2019).
17. K. C. Johnson *et al.*, Single-cell multimodal glioma analyses identify epigenetic regulators of cellular plasticity and environmental stress response. *Nat Genet* **53**, 1456-1468 (2021).
